# Supplementary figures and images for: FURNA: A database for functional annotations of RNA structures
Source: PLoS Biol. 2024 Jul 29;22(7):e3002476. doi: 10.1371/journal.pbio.3002476 (PMC11309384; doi:10.1371/journal.pbio.3002476)

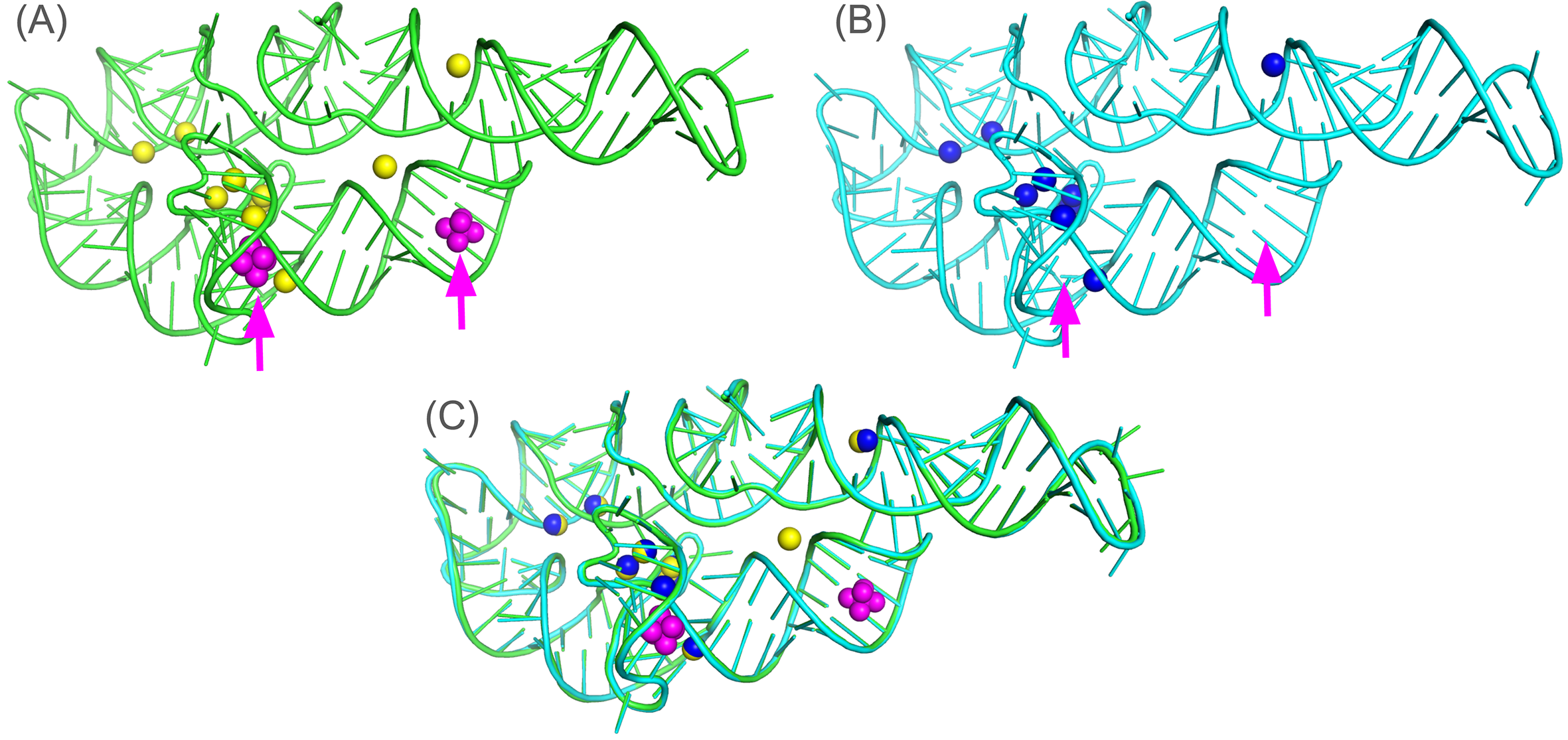

Supplement: S1 Fig — Shown are X-ray structures of group I intron P4-P6 domains from Tetrahymena thermophila in complex with Mg2+ and cobalt hexammine (III). (A) Structure determined with Mg2+ (yellow spheres) and Cobalt hexammine (III) (magenta spheres), PDB 1gid chain A. (B) Structure determined with Mg2+ (blue spheres) only, PDB 6d8o chain A. (C) Overlap of the 2 structures. Magenta arrows in panels (A) and (B) indicate cobalt hexammine (III) binding sites, which are completely different from Mg2+ binding sites. (TIF) [file pbio.3002476.s001.tif]

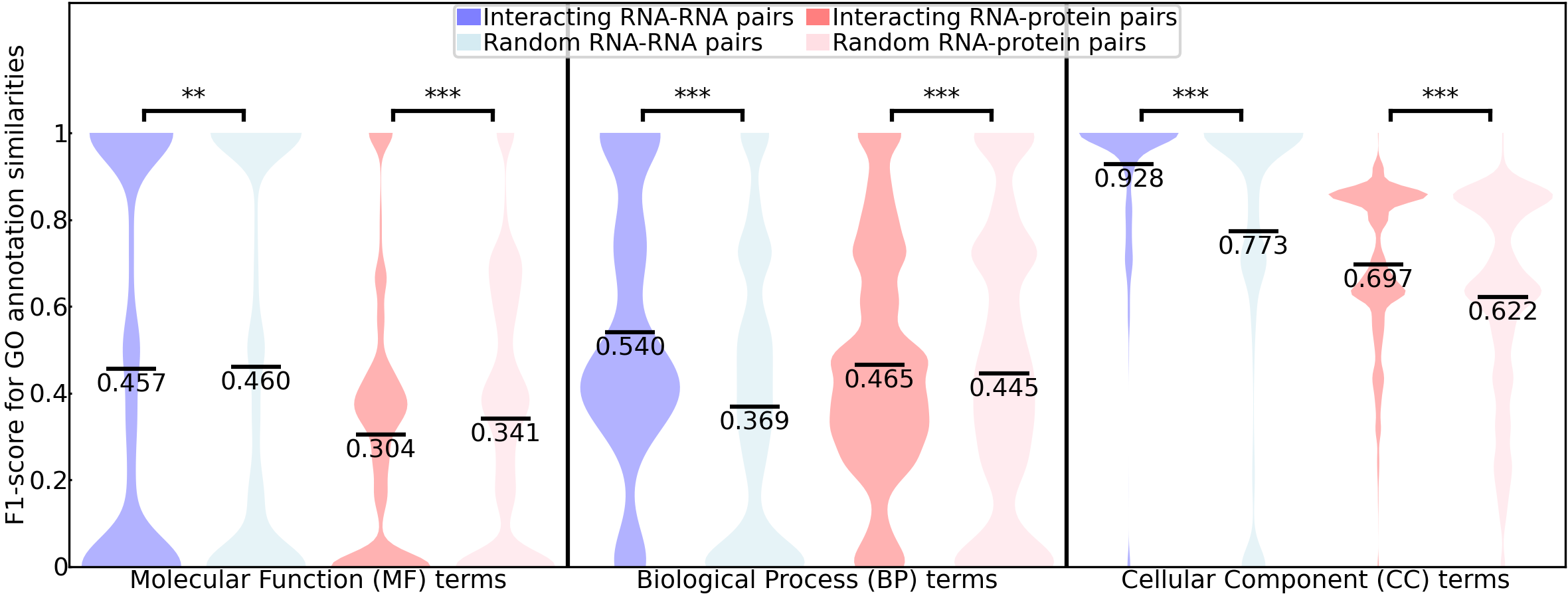

Supplement: S2 Fig — Here, the F1-score of GO annotations between molecule A and molecule B is calculated as: F1=2∙|GOA∩GOB||GOA|+|GOB| Here, GOA and GOB are the set of GO terms (including parent terms) in molecule A and molecule B, respectively. The solid horizontal bars inside each violin show the mean F1-score. The values above the violin plots show the p-value of Wilcoxon rank sum tests between adjacent violins. We observe that just as for proteins, inter-molecular interactions provide a substantial amount of information regarding BP and CC terms, but not for MF terms (as might be expected, as interacting pairs should co-localize in the cell and be involved in the same pathway but will typically not have the same function at the molecular level). P-value <0.001 and 0.01~0.001 are marked by *** and **, respectively. (TIF) [file pbio.3002476.s002.tif]

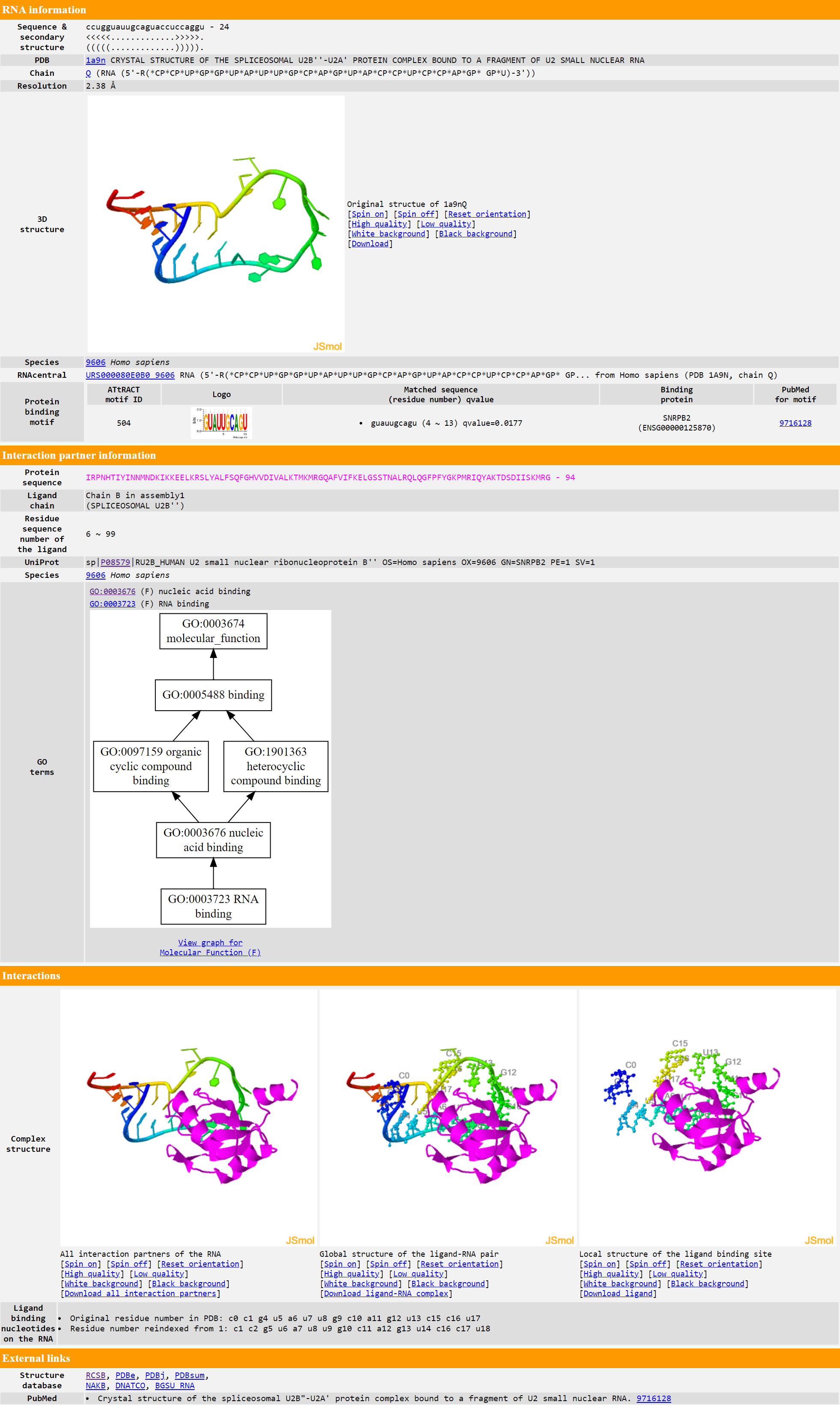

Supplement: S3 Fig — (TIF) [file pbio.3002476.s003.tif]

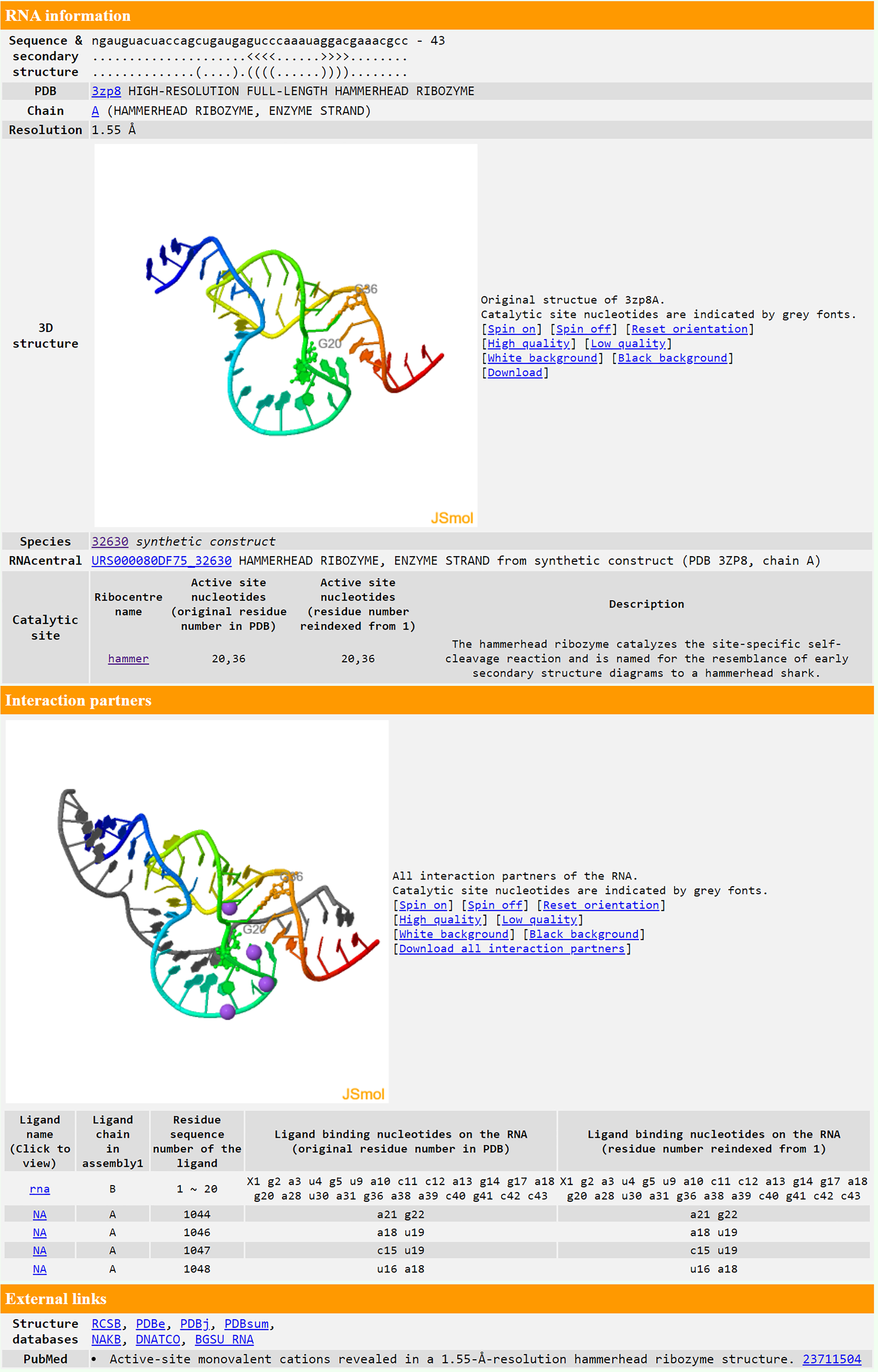

Supplement: S4 Fig — (TIF) [file pbio.3002476.s004.tif]

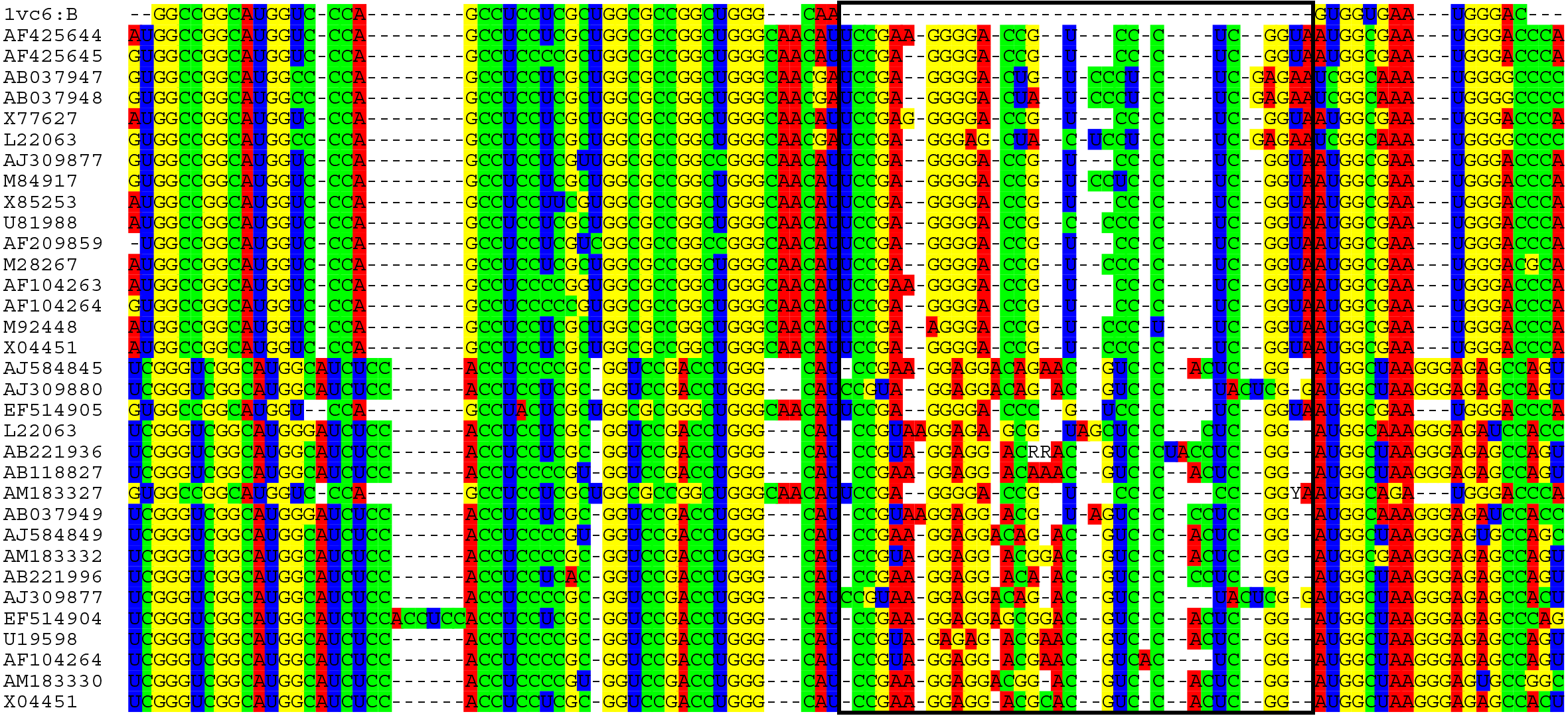

Supplement: S5 Fig — The covariance model region that is absent in the experimental structure is highlighted by a black box. (TIF) [file pbio.3002476.s005.tif]
