# Supplementary material for: FURNA: A database for functional annotations of RNA structures
Source: PLoS Biol. 2024 Jul 29;22(7):e3002476. doi: 10.1371/journal.pbio.3002476 (PMC11309384; doi:10.1371/journal.pbio.3002476)
Supplement: S1 Table — (DOCX) [file pbio.3002476.s006.docx]

**Table S1**. Top small molecule compounds in FURNA, excluding monatomic ions.

| Rank | Ligand ID | Count | 2D diagram | Name |
| --- | --- | --- | --- | --- |
| 1 | OHX | 23168 | 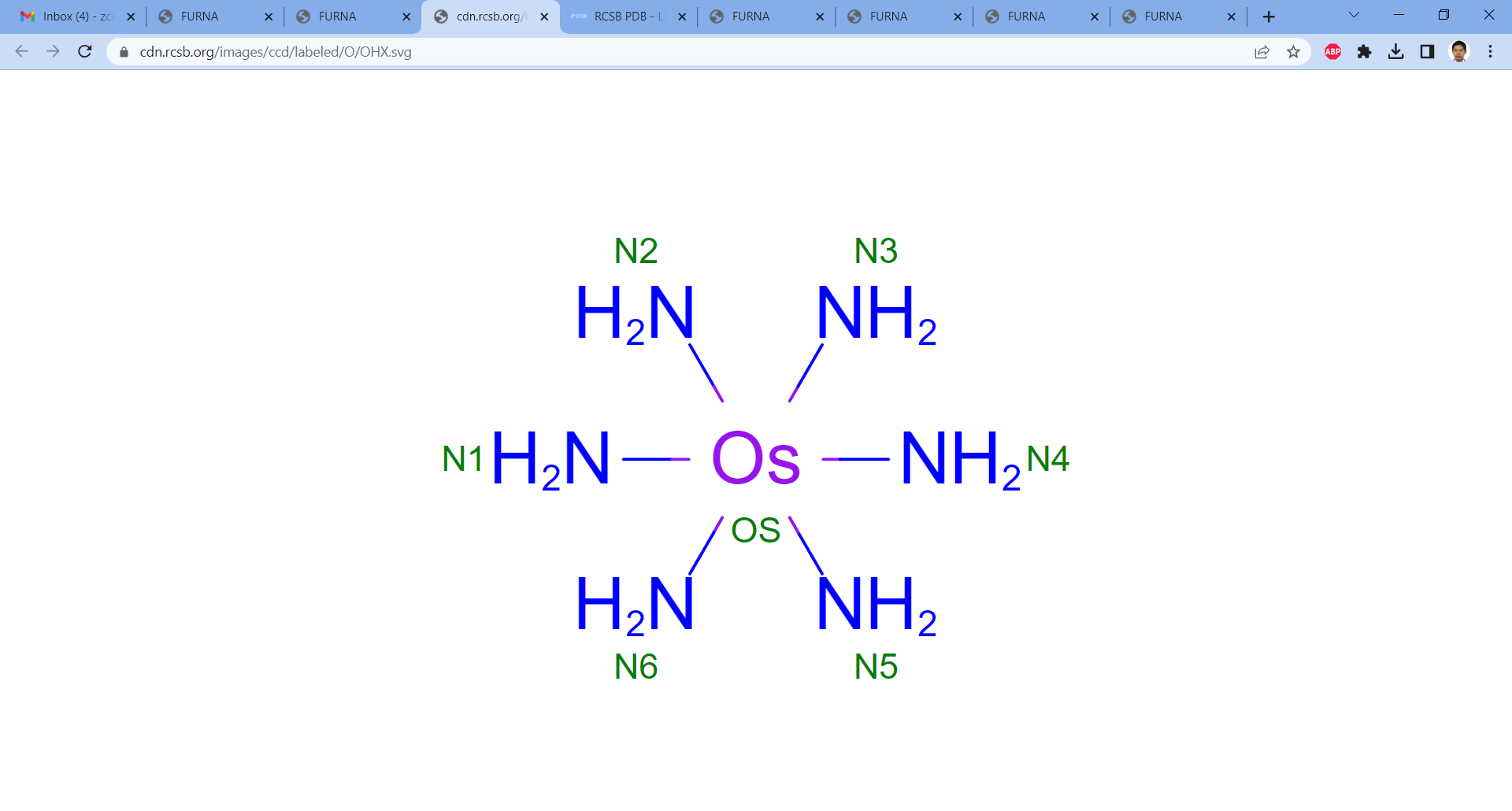 | Osmium (III) hexammine |
| 2 | NCO | 572 | 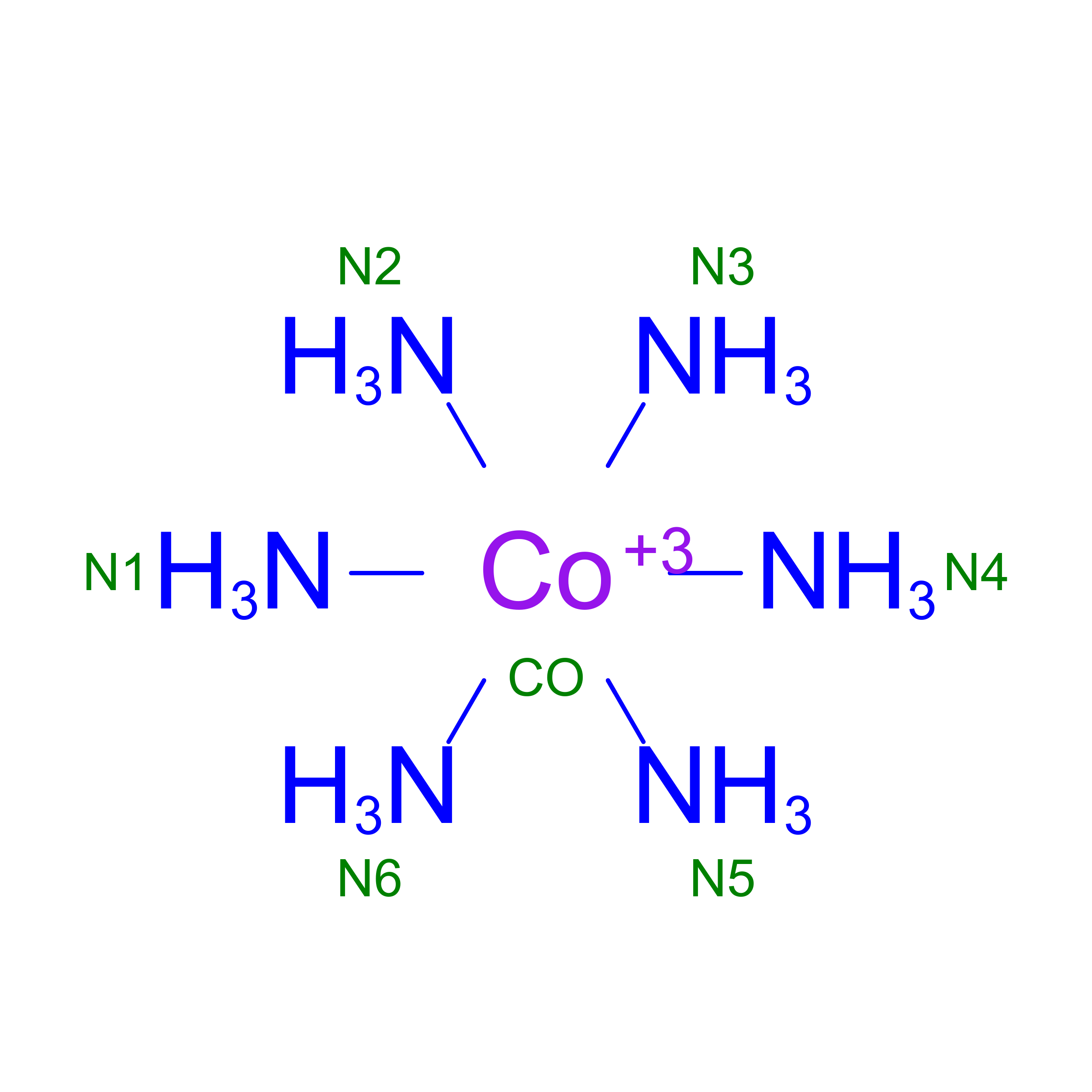 | Cobalt hexammine (III) |
| 3 | SPD | 484 | 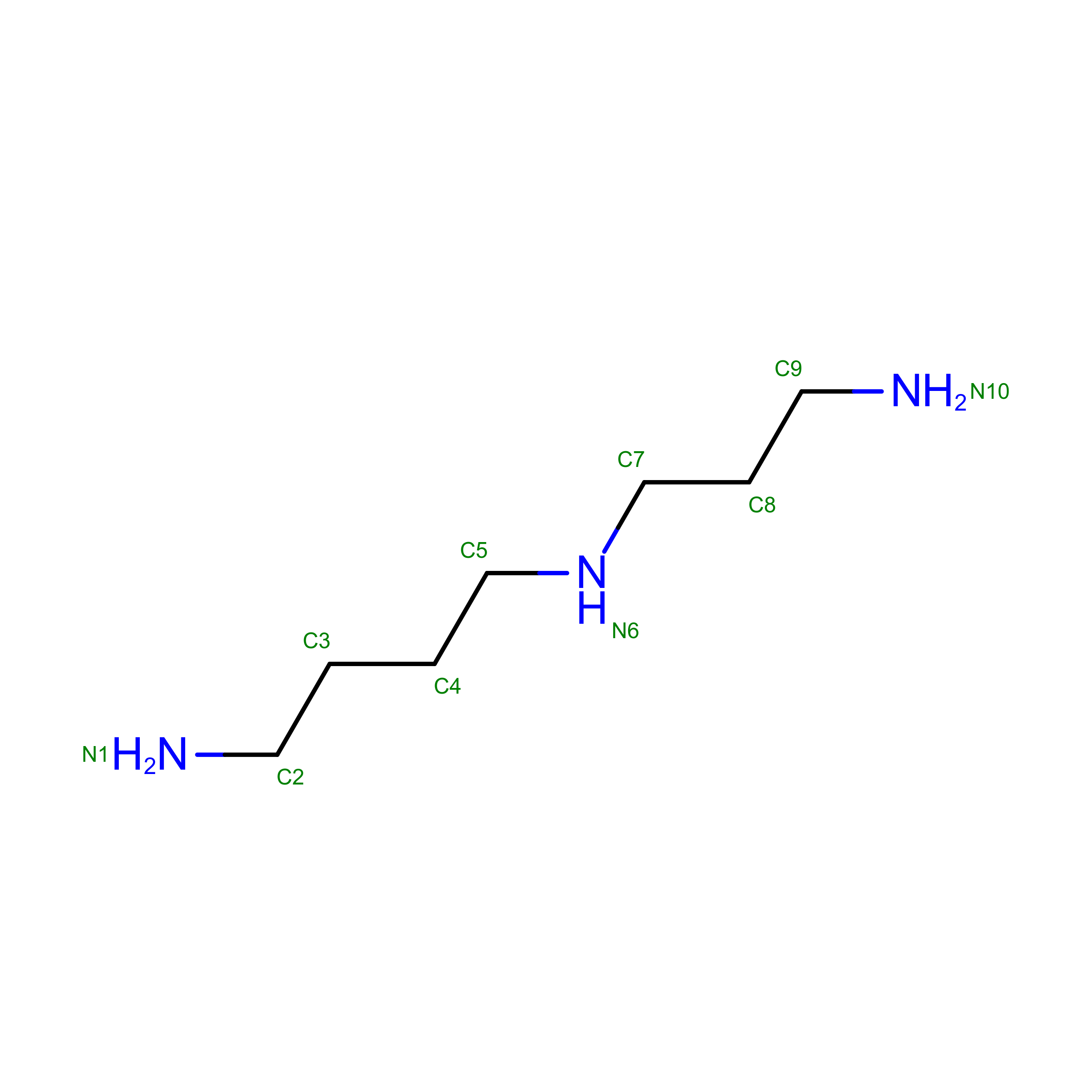 | Spermidine |
| 4 | PAR | 334 | 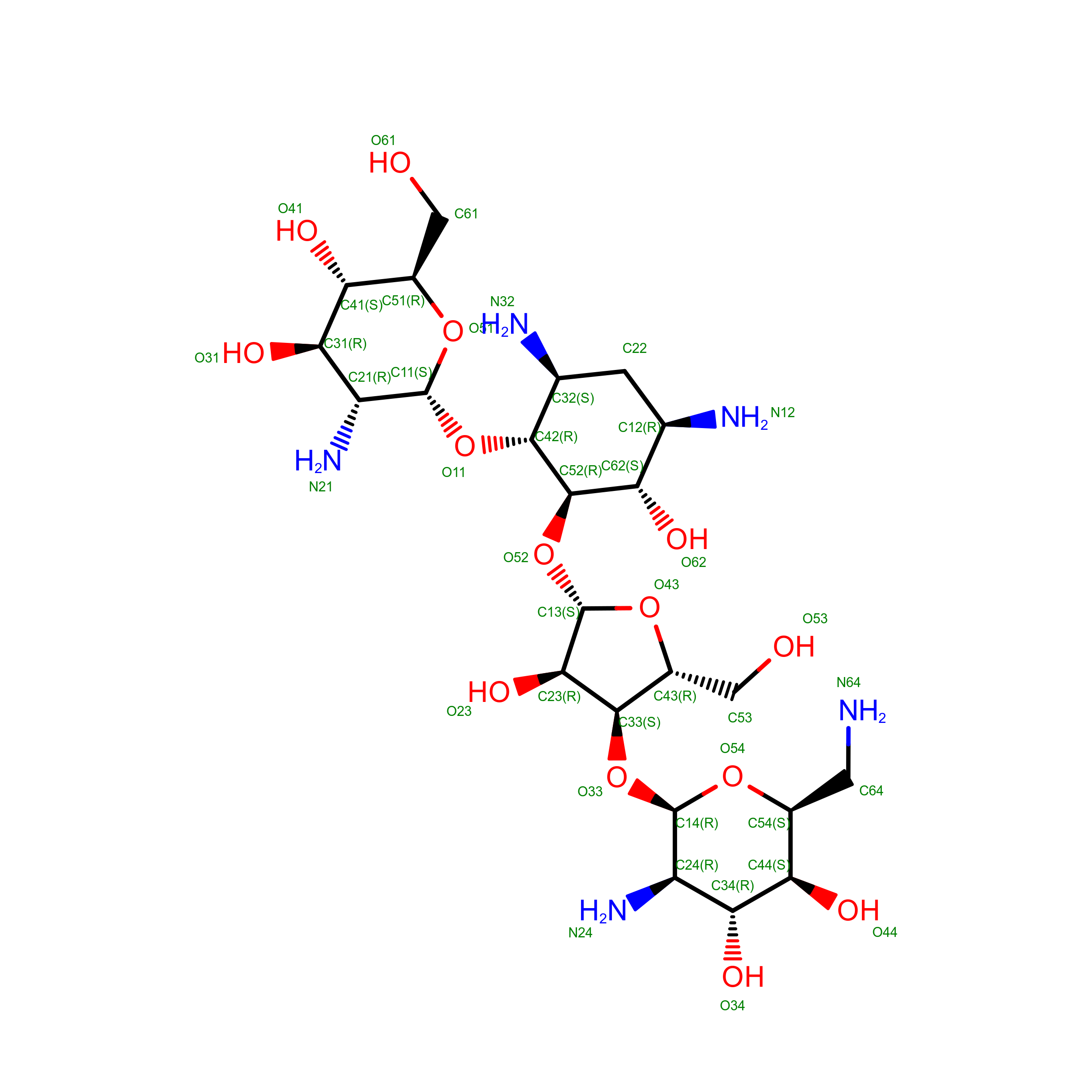 | Paromomycin |
| 5 | IRI | 293 | 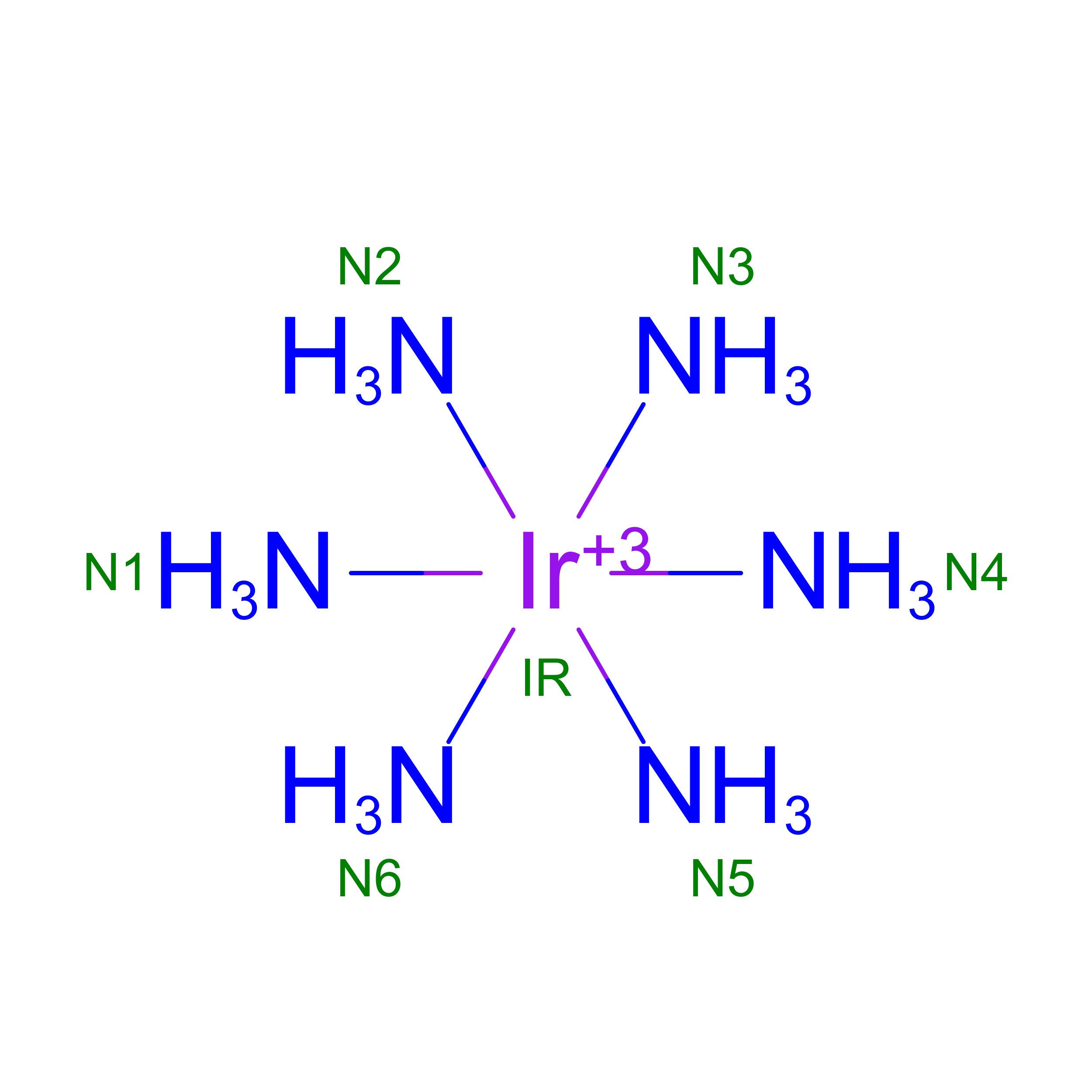 | Iridium hexammine ion |
| 6 | PUT | 256 | 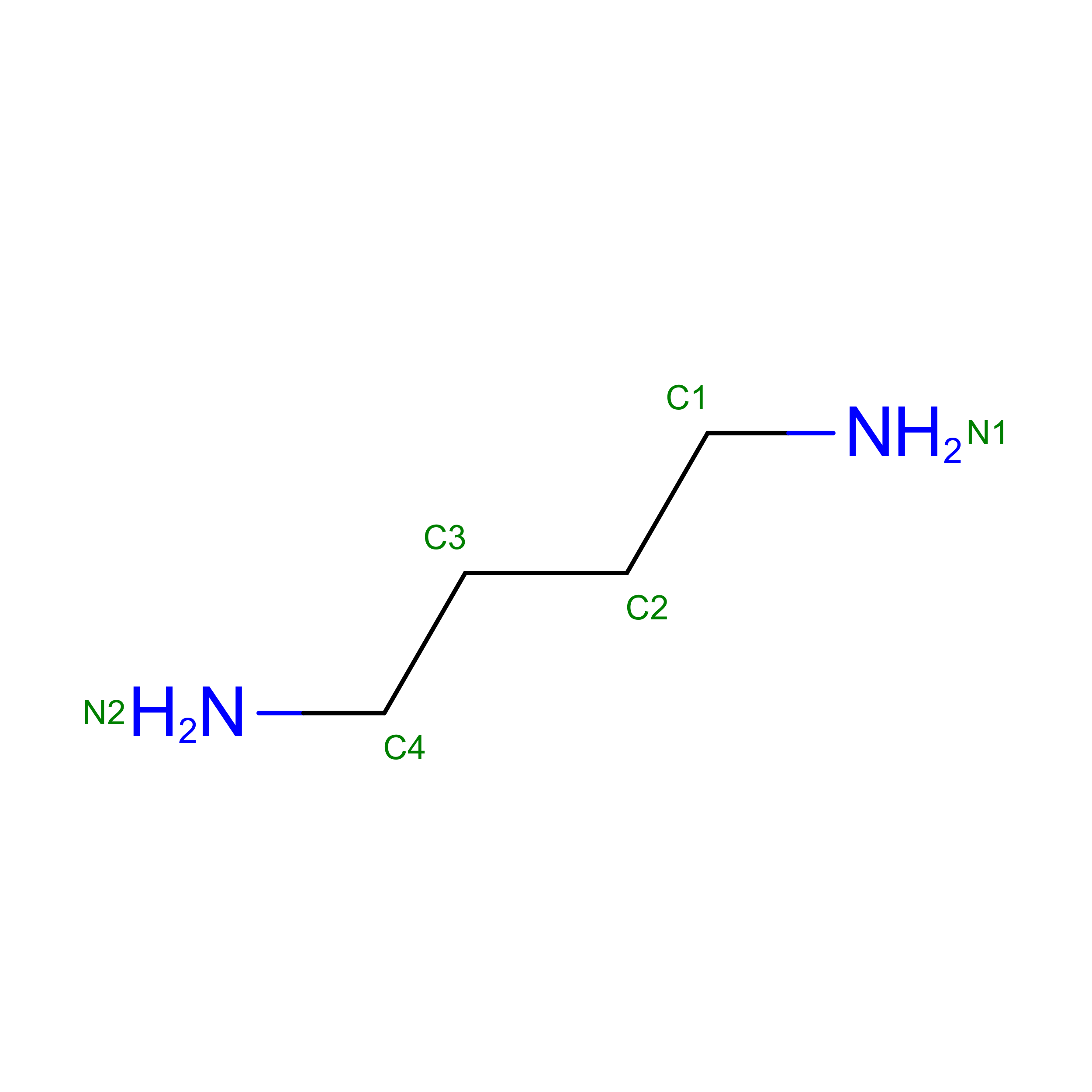 | 1,4-diaminobutane |
| 7 | MPD | 212 | 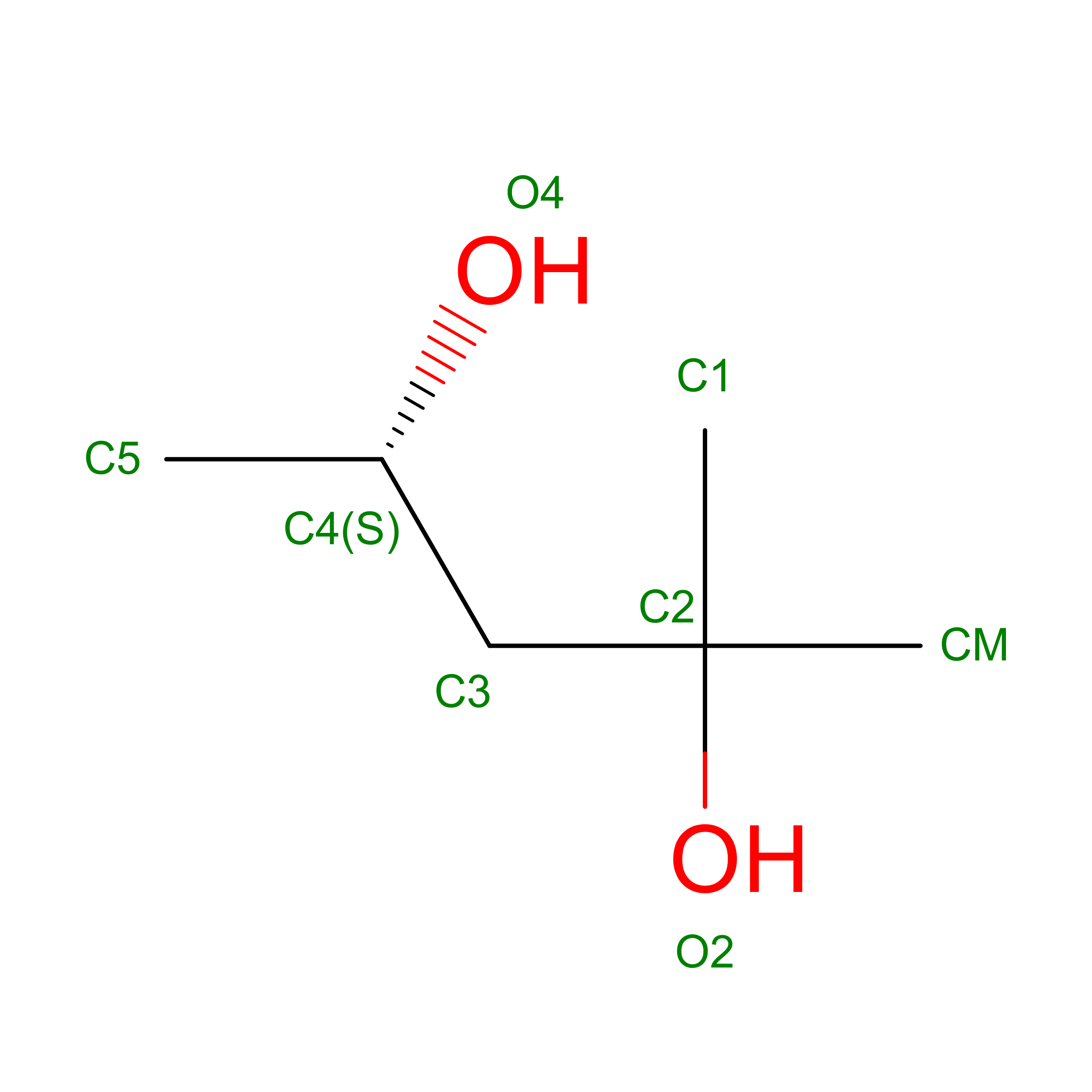 | (4S)-2-methyl-2,4-pentanediol |
| 8 | SPM | 133 | 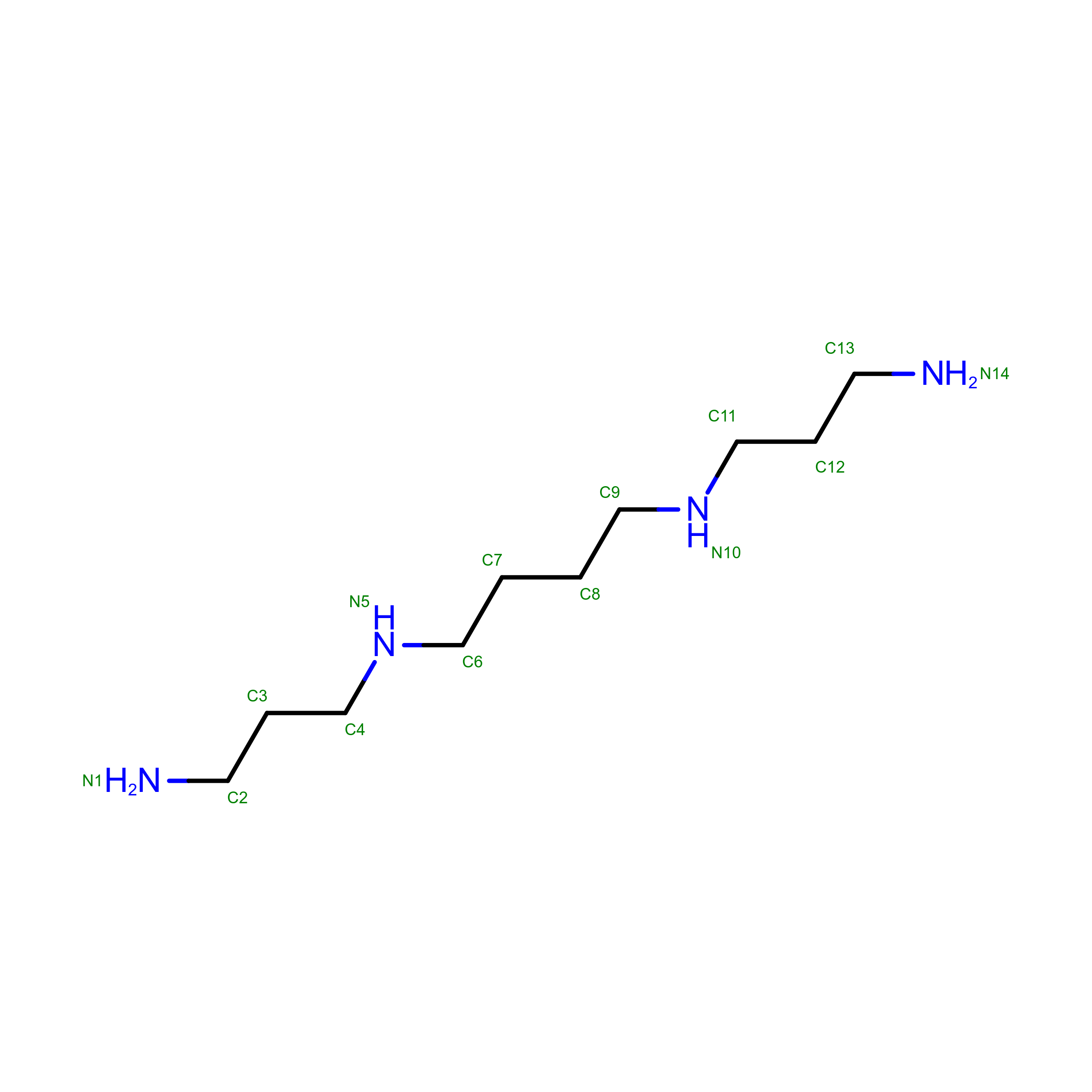 | Spermine |
| 9 | EDO | 110 | 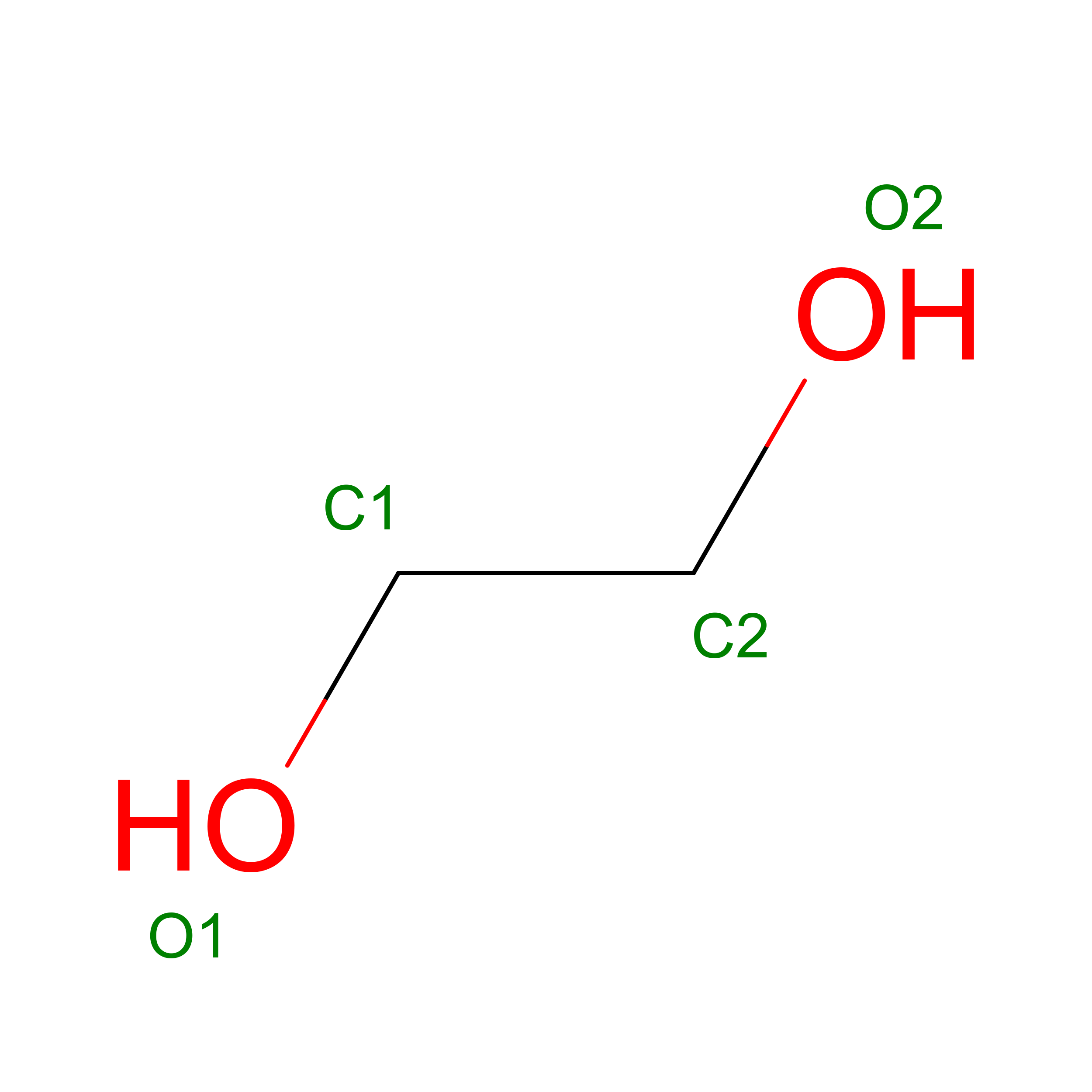 | 1,2-ethanediol |
| 10 | LLL | 98 | 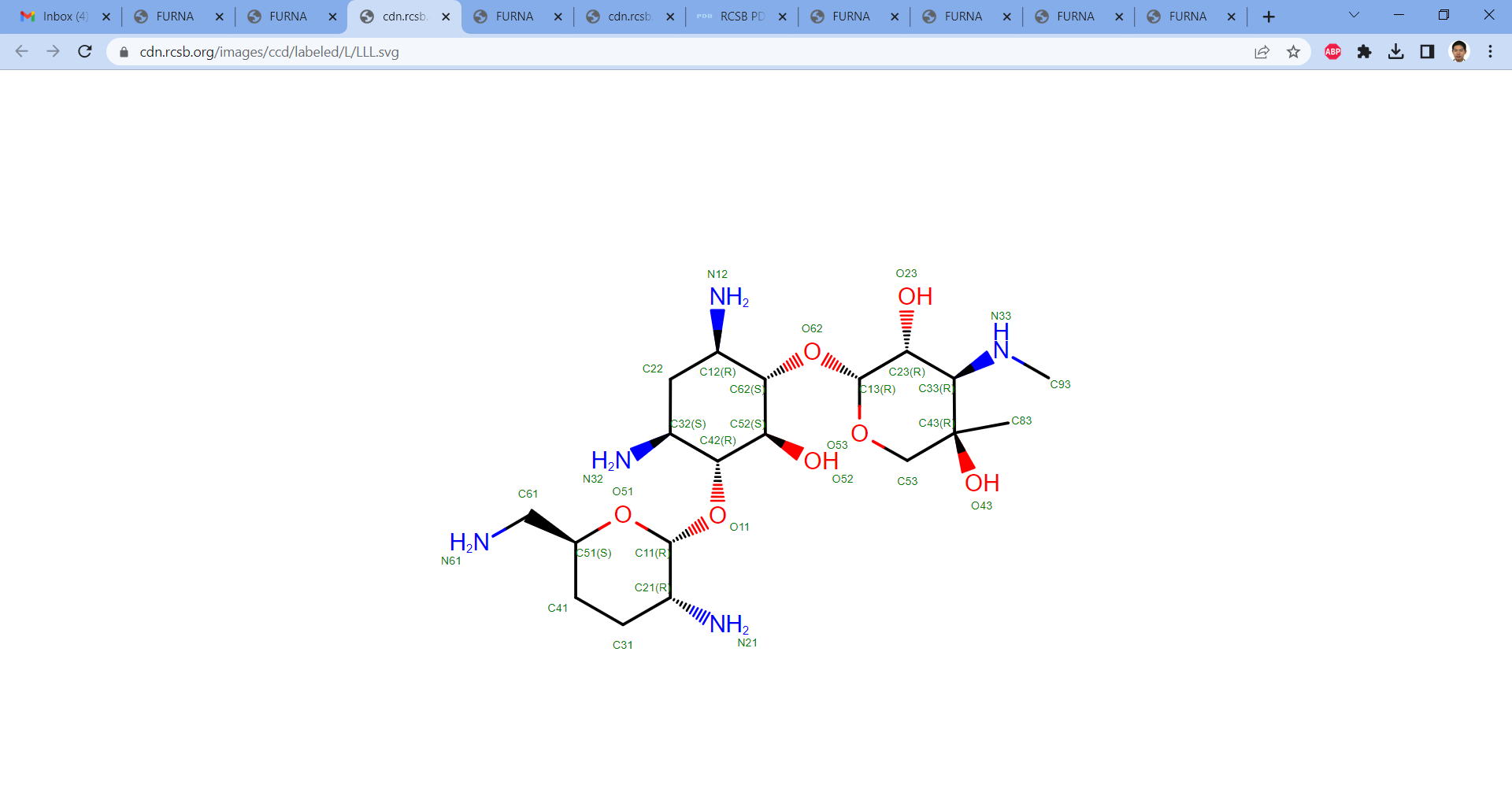 | Gentamicin C1A |
