# Supplementary material for: FURNA: A database for functional annotations of RNA structures
Source: PLoS Biol. 2024 Jul 29;22(7):e3002476. doi: 10.1371/journal.pbio.3002476 (PMC11309384; doi:10.1371/journal.pbio.3002476)
Supplement: S2 Table — (DOCX) [file pbio.3002476.s007.docx]

**Table S2.** The TPP riboswitches used for our case study.

| NCBI accession,  and species | RNA sequence * | Top 5 FURNA hits by BLAST | Top 5 FURNA hits by Infernal |
| --- | --- | --- | --- |
| U00096.3 (position 2185501 to 2185302),  *Escherichia coli* str. K-12 substr. MG1655 | UGCCGUUUUUCCUCGUUUACAACGCGUGCGCUGGACAUUACCAUCCUCCUCUGCGAUUUAUCAUCGCAACCAAACGACUCGGGGUGCCCUUCUGCGUGAAGGCUGAGAAAUACCCGUAUCACCUGAUCUGGAUAAUGCCAGCGUAGGGAAGUCACGGACCACCAGGUCAUUGCUUCUUCACGUUAUGGCAGGAGCAAACU | - 2hoo:A (thi-box riboswitch) - 2gdi:X (TPP riboswitch) - 2hom:A (thi-box riboswitch) - 7tda:A (thiM TPP riboswitch) - 7tzu:A (thiM riboswitch) | - 2hoo:A (thi-box riboswitch) - 2gdi:X (TPP riboswitch) - 7tda:A (thiM TPP riboswitch) - 2hoj:A (thi-box riboswitch) - 7tzu:A (thiM riboswitch) |
| NZ_RYYT01000004.1  (position  386889 to 387088),  *Siccibacter turicensis* | CCCUGGGCUUAGCCCCGGCUACACUACUUUGCUGUGAUUUACUCAUGUGUAAUCACCUGUUUUAUAAGUGAAAUGACUCGGGGUGCCCUUCUUCGUUGAAGGCUGAGAAAUACCCGUAUUACCUGAUCUGGAUAAUGCCAGCGUAGGGAAGUCAGAUGCCUGUCCGGUAUCCCUUCUUCACGCCGGACAGGAGCCUGCUA | - 2hoo:A (thi-box riboswitch) - 2gdi:X (TPP riboswitch) - 7tda:A (thiM TPP riboswitch) - 2hom:A (thi-box riboswitch) - 7tzu:A (thiM riboswitch) | - 2hoo:A (thi-box riboswitch) - 2gdi:X (TPP riboswitch) - 7tda:A (thiM TPP riboswitch) - 2hoj:A (thi-box riboswitch) - 7tzu:A (thiM riboswitch) |
| CP006881.1 (position 1354932 to 1354733),  *Bacillus subtilis* PY79 | UAGGGGUGCUGUUUUGGCUGAGAUAAAGCGCGGAAGAAACGCGCUUUGAUCCCUUAUGACCCGAUCUGGAUAAUACCAGCGUGGGGAAGUGCAGGUUGACCGAAUGGUGUAUUUUUUUGUGCGCUUAAUCGAUCUAUGACUGCAUAUUCCCUUAGGAUAUGCAGUUUUUUAUUUUACCAAAAAAACAGGAGGUCGGAGAA | No BLAST hit found | - 2gdi:X (TPP riboswitch) - 2hoo:A (thi-box riboswitch) - 7tzu:A (thiM riboswitch) - 7td7:A (thiM riboswitch) - 2hoj:A (thi-box riboswitch) |
| CP006881.1 (position 1205283 to 1205483),  *Bacillus subtilis* PY79 | CAAUAUGUAUUCGUUUAACCACUAGGGGUGUCCUUCAUAAGGGCUGAGAUAAAAGUGUGACUUUUAGACCCUCAUAACUUGAACAGGUUCAGACCUGCGUAGGGAAGUGGAGCGGUAUUUGUGUUAUUUUACUAUGCCAAUUCCAAACCACUUUUCCUUGCGGGAAAGUGGUUUUUUUAUUUUCAGAGGGGGAAUGAUUU | No BLAST  hit found | - 7tda:A (thiM TPP riboswitch) - 2hoo:A (thi-box riboswitch) - 2hoj:A (thi-box riboswitch) - 2gdi:X (TPP riboswitch) - 2hom:A (thi-box riboswitch) |

* All RNA sequences are from the 200 nucleotides immediately upstream of the open reading frames for *thiM* (*E. coli* and *S. turicensis*), *ykoF* (the first *B. subtilis* RNA), or *tenA* (the second *B. subtilis* RNA).
