# Supplementary material for: FURNA: A database for functional annotations of RNA structures
Source: PLoS Biol. 2024 Jul 29;22(7):e3002476. doi: 10.1371/journal.pbio.3002476 (PMC11309384; doi:10.1371/journal.pbio.3002476)
Supplement: S3 Table — (DOCX) [file pbio.3002476.s008.docx]

**Table S3.** Annotations of active site nucleotides for the 15 types of ribozymes currently covered by the Ribocentre database.

| **ID** | **PDB chain *** | **Length** | **Rfam** | **Active site nucleotides ^†^** | **Description** |
| --- | --- | --- | --- | --- | --- |
| HDV | 4prf:B | 74 | RF00094 | 100,101,163 (2,3,65) | Hepatitis delta virus (HDV) ribozyme |
| HDV-like | 7qr4:B | 69 | RF00622 | 59 | HDV-like ribozyme |
| Hairpin | 1m5o:B | 92 | RF00173 RF04190 RF04191 | 8,57 | Hairpin ribozymes |
| Hatchet | 6jq5:A | 82 | RF02678 | 1,31,62,63,64 | Hatchet ribozyme |
| Pistol | 6r47:A | 50 | RF02679 | 32,33,40,41,42  (31,32,39,40,41) | Pistol ribozyme |
| RNaseP | 3q1q:B | 347 | RF00009 RF00010 RF00011 RF00030 RF00373 RF02357 RF02375 | 49,50,51,52, 321,322,323 | Ribonuclease (RNase) P |
| Ribosome | 1vqn:0 | 1429 | RF00002 RF02540 RF02541 RF02543 RF02546 | 2284,2285,2485,2486,2487, 2541,2588,2618,2620,2637  (806,807,1002,1003,1004, 1058,1105,1135,1137,1154) | Ribosome large subunit |
| VS-ribozyme | 4r4v:A | 185 | NA | 620,621,638,751  (20,21,38,151) | Varkud satellite (VS) ribozyme |
| glms | 2ho7:B | 123 | RF00234 | 40 (18) | glmS riboswitch |
| groupI | 7ez0:N | 387 | RF00028 | 261,262,263,264,265,266, 306,309,310,311,312  (240,241,242,243,244,245, 285,288,289,290,291) | Group I intron |
| LC-ribozyme | 4p8z:A | 188 | RF01807 | 166,167,168,169 | Lariat capping ribozyme |
| groupII | 4far:A | 390 | RF00029 | 358,359,375,376,377 | Group II intron |
| hammer | 3zd5:A | 42 | RF00008 RF00163 RF02275 RF02276 RF02277 RF03152 | 20,36  (19,35) | Hammerhead ribozyme |
| twister-sister | 5t5a:A | 62 | RF02681 | 7,9,54,55 | Twister sister (TS) ribozyme |
| twister | 4oji:A | 52 | RF03160 | 7,45  (7,43) | Twister ribozyme |

* 4prf:B means PDB 4prf chain B. For the ribosome large subunit, only the last 1429 nucleotides at the 3’ terminal of 1vqn:0 are considered by FURNA, as the full length chain is too long to process (2749 nucleotides) and the first 1320 nucleotides do not contain any nucleotides critical for the catalytic activity,

^†^ Values within the parenthesis are the nucleotide residue numbers after reindexing all residues of the PDB chain starting from 1. If they are the same as the original nucleotide numbers in the PDB file, they are not shown.
