# Supplementary material for: FURNA: A database for functional annotations of RNA structures
Source: PLoS Biol. 2024 Jul 29;22(7):e3002476. doi: 10.1371/journal.pbio.3002476 (PMC11309384; doi:10.1371/journal.pbio.3002476)
Supplement: Attachment — Submitted filename: response_to_reviewers_clean.pdf [file pbio.3002476.s009.pdf]

Response to Reviewer #1

We very much appreciate the comments and suggestions from the Reviewer, especially the suggestion to improve the visualization of ribozyme active sites. Below, we include point-by-point replies to the comments of the Reviewer, where all changes have been highlighted in yellow in the manuscript.

1. **<u>Reviewer #1 commented:</u>**

> ***The manuscript by Zhang and Freddolino describes the development of a database called "FURNA" (Functions of RNA) that seeks to provide a comprehensive annotation of RNA structures in the PDB database. The functional components of the RNA that the authors focus upon are the RNA's interactions with ligands including small molecules, ions, proteins and other DNAs. In the database, the authors provide an easy-to-use search interface with a number of descriptors that enables a user to readily find a particular class of RNAs or an individual sequence, or a set of RNAs that interact with a specific ligand (probably the most useful). This list of hits then includes a set of links including the PDB, Rfam and a GO database. This functional annotation of RNA structures is unique to RNA databases and thus should be a useful tool for many RNA researchers.***
>
> ***Overall the manuscript is mostly well-written and provides a good walk-through of their database and its features.***

We appreciate the precise summary and positive comments from the Reviewer on the work.

2. **<u>Reviewer #1 commented:</u>**

> ***However, I do have a few suggestions to improve the manuscript and database as detailed below.***
>
> ***1. line 48-49. RNA quarternary structure describes a set of RNA-RNA interactions (akin to proteins; refer to an excellent review by Jones and Ferre-D'Amare on this topic) and not RNA-ligand interactions. Please rephrase.***

In the sentence the Reviewer mentioned, the "quarternary structure" indeed refers to RNA-RNA interactions and RNA-protein interactions represented by the biological assembly file. We have clarified this in the revised sentence, which can be found at the end of page 1:

> FURNA determines RNA-ligand interactions based on the biological assembly (i.e., biounit). This approach situates RNA-ligand interactions within the context of its quaternary structure (i.e., the RNA's interaction with nucleic acid and protein partners).

3. **<u>Reviewer #1 commented:</u>**

> ***2. Line 68-70. I would consider osmium hexamine, iridium hexamine and cobalt hexamine (all frequent ligands in RNA structures) to be ionic compounds as they are all metallic ions that have a set of inner sphere coordinations. These amines are the same***

as waters in Mg(2+) or other transition metals, but much more slowly exchanging, which is why we observe them with high occupancy in the density map.

We thank the Reviewer for making the comment. In the revised Results section at page 3, we explained why the FURNA database does not classify them into metal ions with a supplementary illustration.

For small molecule ligands that are not metal ions, the two most frequent compounds are osmium (III) hexammine and cobalt hexammine (III) (**Table S1**), which are crystallization additives used to determine the RNA structure by multiwavelength anomalous diffraction (MAD) phasing [27]. Although these compounds are metal-containing coordination complexes with positive charges, FURNA does not consider them to be metal ions because they are not natural ions, do not bind to natural binding sites biologically relevant metal ions (**Figure S1**), and do not consist solely of a single metal atom.

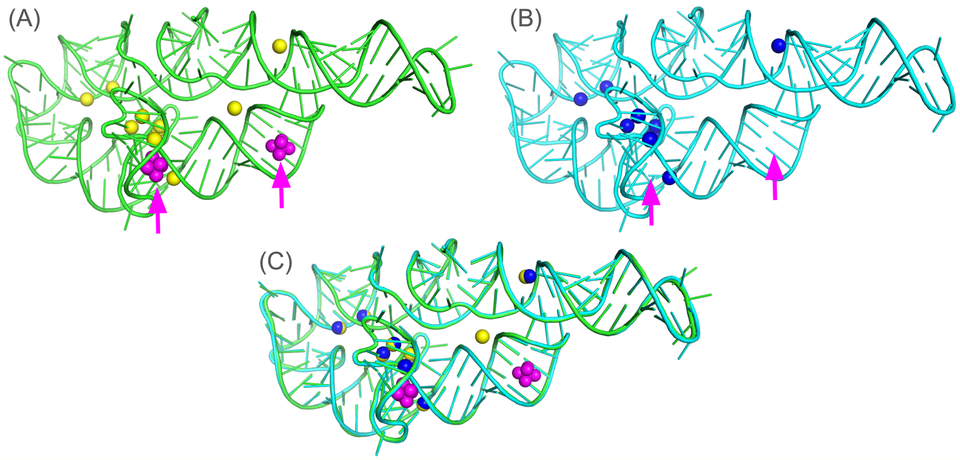

Figure S1. Example of binding sites for biologically relevant cations versus phasing additives. Shown are X-ray structures of group I intron P4-P6 domains from *Tetrahymena thermophila* in complex with Mg^{2+} and cobalt hexammine (III). (A) Structure determined with Mg^{2+} (yellow spheres) and Cobalt hexammine (III) (magenta spheres), PDB 1gid chain A. (B) Structure determined with Mg^{2+} (blue spheres) only, PDB 6d8o chain A. (C) Overlap of the two structures. Magenta arrows in panels (A) and (B) indicate cobalt hexammine (III) binding sites, which are completely different from Mg^{2+} binding sites.

4. <u>Reviewer #1 commented:</u>

3. Lines 160-165. I found this whole paragraph to be confusing. I think that I understand the point the authors are conveying (the ability of FURNA to find examples of weakly related RNAs to the search term), but how this idea is conveyed in the paragraph makes it difficult to determine what the authors specifically did in their search. Further, would these weaker hits be present in Rfam in their list of RNA structures? (As an RNA scientist, I would never use BLAST to find other representatives of the RNA).

We thank the Reviewer for pointing out the issue. In the revised "Case study on TPP riboswitches" section at page 7, we have clarified the procedures used in the case study, and acknowledge that the *B. subtilis* version of TPP riboswitches are present in Rfam and RNAcentral, while still highlighting the additional utility of FURNA in providing another

avenue for identifying that information. Moreover, we clarify our search procedure, where we use the 200 nt upstream of the target gene's ORF as the query sequence to perform the BLAST and Infernal searches.

> Case study on TPP riboswitches
> To illustrate FURNA's utility in RNA function annotation, we conducted a case study involving the TPP (thiamin pyrophosphate) binding riboswitches, also known as the THI element or Thi-box riboswitch. This well-known family of riboswitches binds to thiamine pyrophosphate (TPP) to regulate the expression of its downstream gene [36, 37]. In *Escherichia coli*, one such riboswitch is located upstream of the Hydroxyethylthiazole kinase (*thiM*) coding sequence [38, 39] (**Fig. 3**, **Table S2**). We used the 200 nucleotides immediately upstream of the *thiM* open reading frame as the query sequence to search FURNA. Unsurprisingly, both BLAST (**Fig. 4A**) and Infernal (**Fig. 4B**) searches of the *E. coli* TPP riboswitch through FURNA return hits for many TPP riboswitches, including those from *E. coli*. Similar results can be obtained by searching the region upstream of the *thiM* gene of *Siccibacter turicensis*, which also belongs to the Enterobacteriaceae family (**Table S2**).
>
> Based on gene function and the general prevalence of Thi-box riboswitches, we suspected the presence of riboswitches at several locations in *Bacillus subtilis*, e.g., one situated upstream of the coding sequence of the HMP/thiamine-binding protein (*ykoF*) and the other situated upstream of the aminopyrimidine aminohydrolase (tenA, **Table S2**). Indeed, the *tenA* riboswitch has been previously reported [40], whereas a riboswitch upstream of *ykoF* has not, to our knowledge, been previously reported in the literature, although its presence is indicated in the RNAcentral (https://rnacentral.org/rna/URS000005CA97) and Rfam (https://rfam.org/family/RF00059) databases. When using FURNA to perform a BLAST sequence search of the putative *B. subtilis* TPP riboswitches (i.e., the 200 nucleotides immediately upstream of the *ykoF* and *tenA* open reading frames), no hits are returned, not to the *E. coli thiM* riboswitch. This outcome is not unexpected considering *E. coli* and *B. subtilis* are gram-negative and gram-positive bacteria, respectively, and have evolved separately for billions of years. In contrast, a sensitive Infernal search using either of the potential *B. subtilis* TPP riboswitches does yield hits to other TPP riboswitches, including the *E. coli thiM* riboswitch (**Fig. 5**). These findings highlight FURNA's capability for function annotations of low-homology RNAs using its sensitive sequence search option, providing a unified interface for obtaining functional information on a new RNA of interest.

5. Reviewer #1 commented:

> ***In looking at the database, I found a few things I think could be improved.***
>
> ***1. In a search on a riboswitch that binds a ligand, the page that yields different structures is very helpful and is well-annotated. I particularly like the third view that yield details of the RNA that directly interact with the ligand. However, when I tried to download the coordinates of this structure, all I got was the ligand (not useful). Being able to download these coordinates I think is crucial, because viewing in the database viewer is very limited as compared to Chimera or PyMol. Considering revising this feature.***

We thank the Reviewer for raising this concern, which is probably caused by our unclear writing. In fact, the webpage indeed provides the download link for the coordinates of the ligand in complex with the RNA. Specifically, in the "Interactions" section of the webpage, three structure applets correspond to all interaction partners of the RNA, structure of the

ligand-RNA pair, and the local structure of the ligand binding site. There are three download links under these applets. The Reviewer is probably using the rightmost link for "Download ligand". If the Reviewer needs the RNA in complex with the ligand, they should use the two links on the left, i.e., "Download all interaction partners" or "Download ligand-RNA complex", as shown within the red box in the following figure.

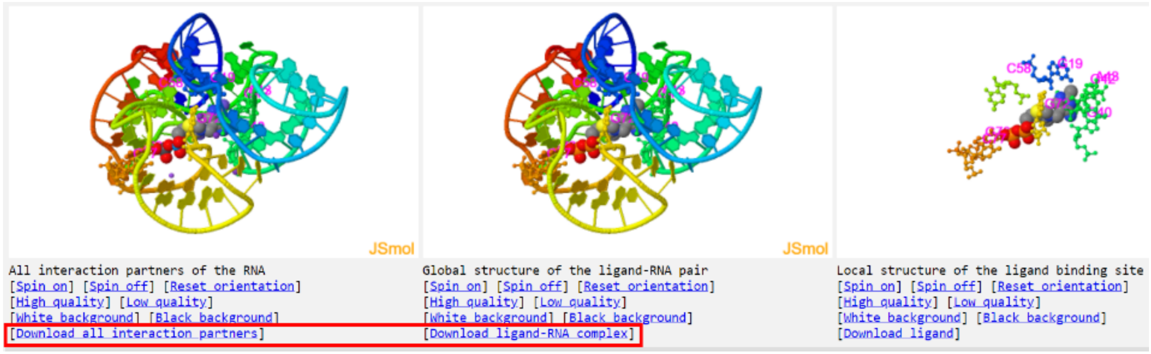

To explain this point, we have revised the Figure 3 caption accordingly:

> **Fig 3. Excerpt of an individual FURNA web page for a small molecule-RNA interaction.**
> Interaction between thiamine diphosphate and TPP riboswitch (PDB 2gdi chain X, https://seq2fun.dcmb.med.umich.edu/furna/pdb.cgi?pdbid=2gdi&chain=X&lig3=TPP&ligCha=X&ligIdx=1). The structures of the RNA with all interaction partners, the RNA with only the ligand of interest, and the ligand itself without the RNA can be downloaded through the links at the bottom of the "Interaction" section.

6. <u>Reviewer #1 commented:</u>

> ***2. Searching on the hammerhead ribozyme (5EAQ) yields a very confusing description of the structure. Specifically, the authors look at the RNA from the perspective of the enzyme strand and consider the substate strand a ligand. This leads to some strange views of the RNA structure as just the enzyme strand (top view), which really does not reflect the biological assembly.***
>
> ***Further, the most important aspect of these RNAs is the catalytic pocket and the nucleotides surrounding the cleavage site—a view not given. Thus, this makes this database really not very useful for catalytic RNAs and understanding the function of their active sites. This issue is rife throughout the database for catalytic RNAs.***

The webpage for the hammerhead ribozyme mentioned by the Reviewer (https://seq2fun.dcmb.med.umich.edu/furna/pdb.cgi?pdbid=5eaq&chain=A) not only shows the structure of the enzyme strand itself, but also shows the enzyme strand in complex with the substrate strand under the Interaction section (See the following screenshot). Here, the enzyme strand is shown in rainbow color (blue to red for 5' to 3' terminus) while the substrate strand is shown in grey.

**PDB**	5eaq Two active site divalent ion in the crystal structure of the hammerhead ribozyme bound to a transition state analog-Mn2+
**Chain**	A (RNA (48-MER))
**Resolution**	3.201 Å
**3D structure**	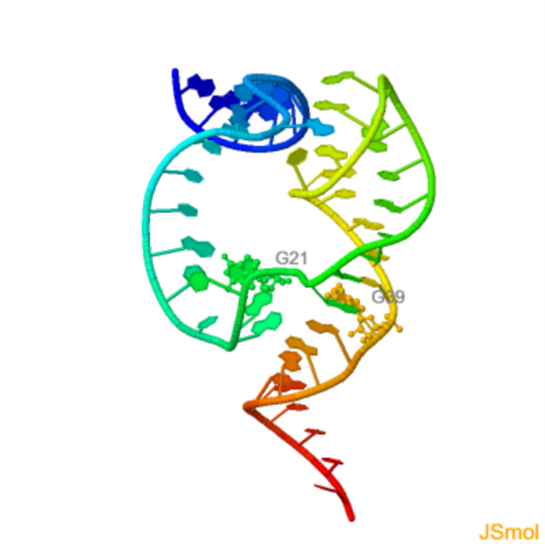 Original structue of 5eaqA. Catalytic site nucleotides are indicated by grey fonts. [Spin on] [Spin off] [Reset orientation] [High quality] [Low quality] [White background] [Black background] [Download]
**Species**	32630 *synthetic construct*
**RNAcentral**	URS0000868531_32630 RNA (48-MER) from synthetic construct (PDB 5DQK, chain A)
**Rfam families**	RF00008 Hammerhead ribozyme (type III)

Catalytic site

Ribocentre name	Active site nucleotides (original residue number in PDB)	Active site nucleotides (residue number reindexed from 1)	Description
hammer	21,39	21,39	The hammerhead ribozyme catalyzes the site-specific self-cleavage reaction and is named for the resemblance of early secondary structure diagrams to a hammerhead shark.

Interaction partners

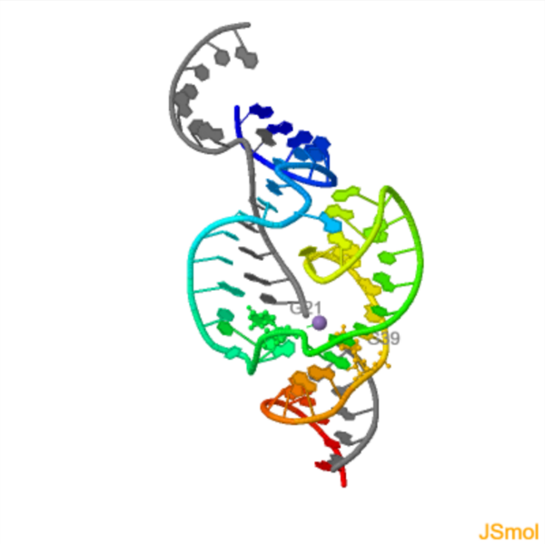

All interaction partners of the RNA.
Catalytic site nucleotides are indicated by grey fonts.
[Spin on] [Spin off] [Reset orientation]
[High quality] [Low quality]
[White background] [Black background]
[Download all interaction partners]

.

We agree with the Reviewer that catalytic site annotation would be very useful. As shown by the above screenshot, the new version of FURNA highlights the catalytic site annotated using grey fonts based on the Ribocentre database. We have added a section “Catalytic site annotation” in the Materials and Methods section at page 9 to explain how this is achieved.

We believe that our additions, prompted by this reviewer comment, substantially expand the utility of FURNA for providing actionable information on ribozymes. At the same time, at present Ribocentre is admittedly limited in its coverage, and we are only able to provide rich ribozyme annotations for cases covered in Ribocentre. We will continue to maintain and update FURNA's inclusion of these annotations, and will also integrate any additional appropriate ribozyme databases that are developed over time; we expect this to be a continuing area of advancement in the community.

Catalytic site annotation
The nucleotides at the active site of ribozymes are annotated based on the Ribocentre [42] database, which is to our knowledge the only curated database for ribozyme active sites available as of this writing. Among all 21 types of ribozymes collected by Ribocentre, detailed active site information on the RNA 3D structure is available only for a subset of 15 types of ribozymes (**Table S3**). For each of these 15 types of ribozymes, active site nucleotides are annotated for only one representative experimental structure. To extend the Ribocentre database annotation to all members of these 15 ribozyme types, we implemented a template-based approach using US-align [43]. Briefly, the representative experimental structure of a ribozyme type is used as the query by US-align to search through all FURNA RNA chains that share the same Rfam families as the target ribozyme type (**Table S3**). FURNA RNAs with significant structure similarity (TM-score≥0.45 [44]) to the query RNA are collected. Additionally, if the query ribozyme has <100 nucleotides, all FURNA RNAs without Rfam families are also searched by US-align to get similar FURNA RNAs with TM-score≥0.45, as the covariance models of the Rfam families for short ribozymes may not match the FURNA RNA sequence with significant E-values as shown in the HDV ribozyme example mentioned in the previous paragraph. Nucleotides on the FURNA RNAs that are aligned to the active sites on the query ribozyme structure are marked as putative active sites.

.

Response to Reviewer #2

We very much appreciate the comments and suggestions from the Reviewer. Below, we include point-by-point replies to the comments of the Reviewer, where all changes have been highlighted in yellow in the manuscript.

1. **<u>Reviewer #2 commented:</u>**

> ***The MS "FURNA: a database for function annotations of RNA structures" describes a newly built database that is filling up so far empty niche of functional annotations of experimentally determined RNA structures. I actually enjoyed reading the MS, it is well written and technically sound, the authors correctly overview the existing structural databases.***
>
> ***I have no serious comments to its publication, just a few notes.***
>
> ***I appreciate that the authors went into trouble with generating symmetry-built biological units.***

We thank the Reviewer for the positive comments.

2. **<u>Reviewer #2 commented:</u>**

> ***The statement "DSSR only functions when the nucleobase of the nucleotide is fully atomic and its RMSD to the standard nucleobase conformation is less than 0.28 Å." On pg. 9 is unclear. What is "standard nucleobase conformation"? In general, I would warn agains using DSSR structural classifications, e.g., its base par assignments are very unreliable, base morphology often sheer nonsense.***

When identifying nucleobases, DSSR uses standard nucleobase conformations collected from the Arnott 1999 citation, which is now added to the revised manuscript:

> Conversely, DSSR only functions when the nucleobase of the nucleotide is fully atomic and its RMSD to the standard nucleobase conformation [50] is less than 0.28 Å.
>
> 50. Arnott S. Polynucleotide secondary structures: an historical perspective. Oxford University Press Oxford, UK; 1999. P. 1-38.

We recognize that DSSR does not always give correct base pair assignments. This is why we include a complementary algorithm (CSSR).

3. **<u>Reviewer #2 commented:</u>**

> ***I would suggest to use Mol* instead of jsmol as it speeds up rendering of large structures but I do understand difficulties with poorly documented Mol* software.***

We thank the Reviewer for the suggestions. We used Jsmol rather than Mol* due to technical limitations of some web browsers, in order to maximize compatibility of our database. A single FURNA webpage needs to show four structure applets. While a WebGL-based applet such as Mol*, 3Dmol or NGL has no difficulty loading one instance of the applet, older versions of web browsers sometimes have difficulty loading multiple

instances of WebGL applet. Although this is an infrequent issue of WebGL, we still prefer Javascript-based applet (JSmol) for maximum compatibility with old browsers.

4. **Reviewer #2 commented:**

> ***The following suggestion is much stronger: use mmCIF instead of PDB format as the the latter is obsolete while the former opens up opportunities to add your own annotations to individual flat files in form of custom-built mmCIF categories.***

We thank the Reviewer for the suggestion. For source data, FURNA always identifies RNA-ligand interactions from mmCIF file rather than PDB files from the PDB database. On the other hand, for web page display, we use the PDB format when possible, mainly due to the limited disk space available on our web server and the near-universality of the PDB format despite its age. We currently have no need to add custom mmCIF categories, although in the future we may update FURNA to use mmCIF files if we would make use of any of the features that they enable. We now justify the choice of both file formats in the Web Interface section at page 4:

> These individual pages include the 3D structures of the RNA chain on its own, the full biological assembly, the RNA-ligand pair, and the local structure of the ligand binding site. These are displayed via four JSmol [32] applets, where the second applet uses the mmCIF-formatted structure while the remaining three applets use PDB format structures. The reason for this difference is that the full biological assembly may contain more than 99,999 atoms and/or 62 chains, making it impossible to be represented in PDB format [33]. On the other hand, an individual RNA-ligand pair can always be represented by a PDB file, which has a smaller file size than an mmCIF file [34] and is compatible with more bioinformatics tools [33].

5. **Reviewer #2 commented:**

> ***Minor thing, on page 2 should be "… many proteins, they ARE critical for the folding of many …"***

We thank the Reviewer of the careful proofreading. We have corrected the typo accordingly at page 3.

> Similarly, while potassium ions (K^+) are a simple buffer additive for many proteins, they are critical for the folding of many large RNAs where potassium ions stabilize juxtaposition of nucleotides with large sequence separation by neutralizing charge density [26].

Response to Reviewer #3

We very much appreciate the comments and suggestions from the Reviewer, especially suggestions to improve the web interface and figure legibility. Below, we include point-by-point replies to the comments of the Reviewer, where all changes have been highlighted in yellow in the manuscript.

1. **<u>Reviewer #3 commented:</u>**

> ***FURNA, by Chengxin Zhang and Lydia Freddolino, collects together a large number of data items concerning RNA chains in the Protein Data Bank. The database is updated every week. The data items are helpful to have pre-computed and to have them available in a central place. Basic search of the annotations is available. More sophisticated searches based on sequence and 3D structure are also provided.***

We thank the Reviewer for the precise summary and positive comments.

2. **<u>Reviewer #3 commented:</u>**

> ***Line 20, 16,000 RNA chains, not 1,600.***
> ***Line 63, they are critical***

We thank the Reviewer for the careful proofreading. We have revised the two sentences as follow at page 1 and page 3:

> Advances in experimental RNA structure determination methods, particularly Cryo-EM [1], have resulted in over 16,000 RNA chains being deposited into the Protein Data Bank (PDB) database [2].
>
> Similarly, while potassium ions (K^+) are a simple buffer additive for many proteins, they are critical for the folding of many large RNAs where potassium ions stabilize juxtaposition of nucleotides with large sequence separation by neutralizing charge density [26].

3. **<u>Reviewer #3 commented:</u>**

> ***Figure 1, it would be great if the text was characters that can be copied and not an image that pixelates when you zoom in, or that has visual artifacts that make some lines harder to read.***

While we understand the Reviewer's concern, we unfortunately cannot implement the Reviewer's suggestion to make Figure 1 a vector image. PLoS will always render the images in PNG and TIFF formats in the final online version of the published manuscript. Therefore, even if we submit a vector image (e.g., in EPS format), they will be pixelated by the journal eventually.

Meanwhile, part of the reason for the Reviewer's concern is caused by the Figure's apparent low resolution. This is a side effect from the proof PDF generated by the PLoS online submission system and not reflect the real resolution of the image. In the new

submission, we include the high-resolution source images at 300 dpi, which should make the text in the published version of the figure easily legible.

4. **<u>Reviewer #3 commented:</u>**

   Figure 1, can the website be used to generate these lists, counts, and histograms? If not, it's OK, just be clear.

Yes, the data to draw Figure 1AB are available under section "Database statistics" of the home page of the FURNA website, although the histograms themselves are not available on website. This is now explained by the revised Figure 1 caption.

> **Fig 1. Overall statistics for RNAs and ligand-RNA interactions in FURNA.**
> (A) Pie chart for the breakdown of ligand types in ligand-RNA interactions. (B) Venn graph showing the numbers of RNAs with GO terms in the MF, BP and/or CC aspects. (C) Top GO terms in RNAs and proteins collected by FURNA. Data for panels (A) and (B) are available under the section "Database statistics" from the home page of the FURNA website.

5. **<u>Reviewer #3 commented:</u>**

   Figure 2 is impossible to read on a printed page, and in the PDF it pixelates and is barely legible. In Panel A, it would be sufficient to show one entry from the summary table, at a resolution where it can be read and explained. Panel B could be split into two images, or some section left out.

We thank the reviewer for the important recommendation to modify Figure 2. We now split Figure 2 into two figures and remove some non-essential entries. We also note that the poor resolution of the figures in the PDF provided for review is an artifact of the PLoS review system; the final manuscript will include 300 dpi images of all relevant screenshots.

#	PDB (Resolution)	Length	EC number & GO term	RNAcentral	Rfam	Taxon	PubMed	ATtRACT motif	Ligand & binding nucleotides
1	1na2:A	30	EC:2.7.7.49 RNA-directed DNA polymerase<br>GO:0003720 (F) telomerase activity<br>GO:0007004 (P) telomere maintenance vi...<br>GO:0000333 (C) telomerase catalytic co...	URS000080DF2D	RF00024	*Homo sapiens*<br>9606	12525685	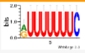<br>s65<br>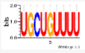<br>58<br>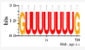<br>s66<br>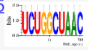<br>122<br>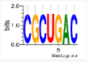<br>1188<br>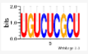<br>1125	
2	1nbs:A<br>(3.15Å)	120	EC:3.1.26.5 ribonuclease P<br>GO:0004526 (F) ribonuclease P activity<br>GO:0008033 (P) tRNA processing<br>GO:0030680 (C) dimeric ribonuclease P ...	URS000080E089	RF00011<br>RF01100	*Bacillus subtilis*<br>1423	12610630		Assembly1<br>PB:A:241 186,219~220<br>PB:A:242 90,132<br>PB:A:244 134,181~182<br>PB:A:249 228~229

Fig 2. Summary table for browsing function annotations in FURNA.

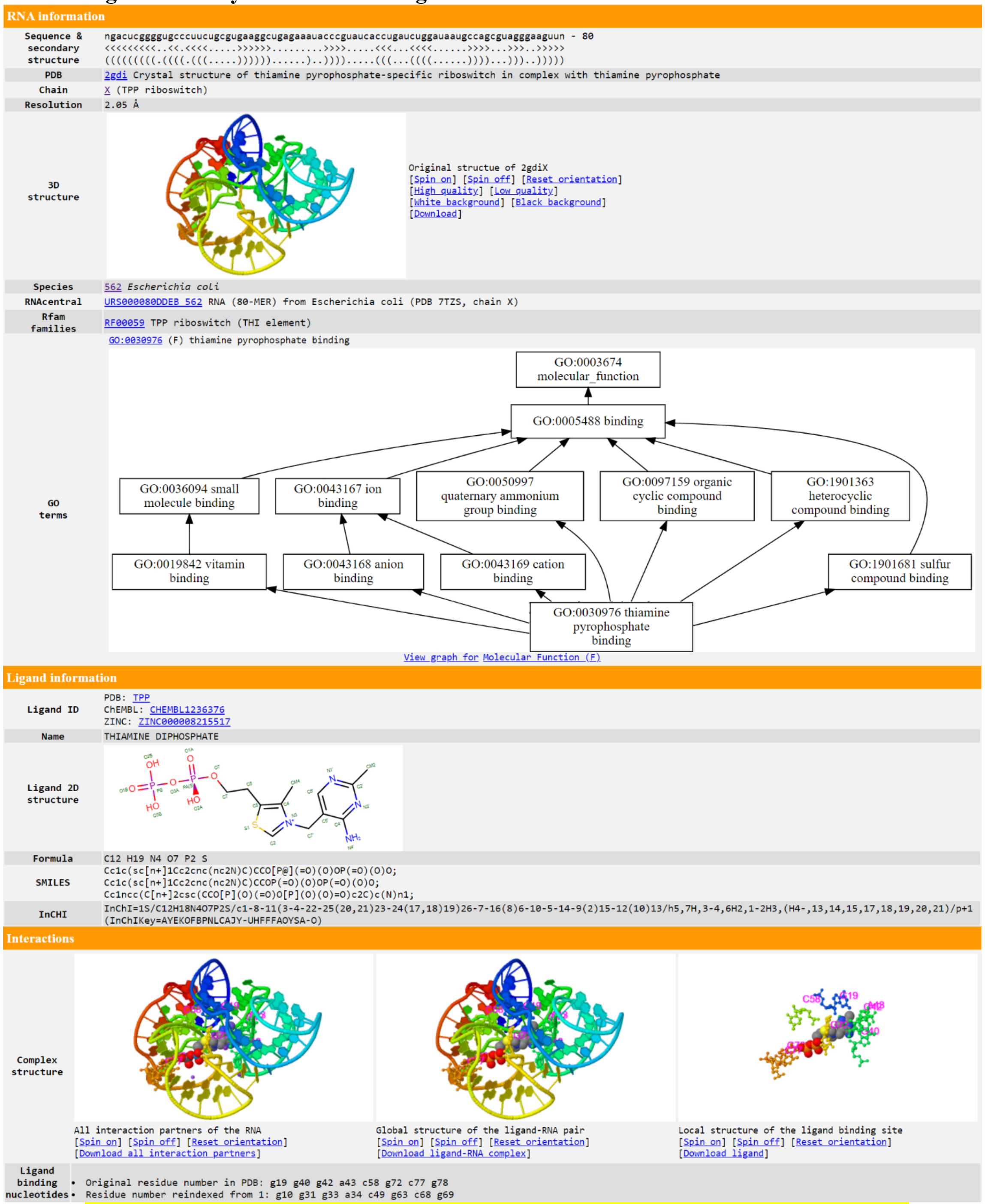

Fig 3. Excerpt of an individual FURNA web page for a small molecule-RNA interaction.

Shown is the FURNA web page for the interaction between thiamine diphosphate and TPP riboswitch (PDB 2gdi chain X, https://seq2fun.dcmb.med.umich.edu/furna/pdb.cgi?pdbid=2gdi&chain=X&lig3=TPP&ligCha=X&ligIdx=1). The structures of the RNA with all interaction partners, the RNA with only the ligand of interest, and the ligand itself without the RNA can be downloaded through the links at the bottom of the "Interaction" section.

6. Reviewer #3 commented:

Figure 3 is very hard to read on a printed page. Reducing the number of lines of output, splitting the figure, could help.

We thank the reviewer for the important suggestion. We now reduce the number of lines from 10 to 5 and split the original Figure 3 into two figures.

(A)

#	Hit	Hit length	Aligned length	Identity (normalized by query)	Identity (normalized by hit)	Identity (normalized by aligned length)	E-value	Homologs to hit
1	2hoo:A Crystal structure of an E. coli thi-box riboswitch bound to benfotiamine; thi-box riboswitch	83	79	0.3950	0.9518	1.0000	2.98e-36	
2	2gdi:X Crystal structure of thiamine pyrophosphate-specific riboswitch in complex with thiamine pyrophosphate; TPP riboswitch	80	77	0.3850	0.9625	1.0000	3.85e-35	2gdi:Y, 7tzs:X, 7tzs:Y, 4nya:A, 4nya:B, 7tzr:X, 7tzr:Y
3	2hom:A Crystal structure of an E. coli thi-box riboswitch bound to thiamine monophosphate; thi-box riboswitch	80	79	0.3800	0.9500	0.9620	3.88e-30	
4	7tda:A Crystal structure of the E. coli thiM riboswitch in complex with thiamine pyrophosphate, manganese ions; thiM TPP riboswitch RNA (80-MER)	80	79	0.3800	0.9500	0.9620	3.88e-30	7tdc:A, 7tdb:A, 4nyd:A, 4nyb:A, 4nyc:A
5	7tzu:A Crystal structure of the E. coli thiM riboswitch bound to 1-(4-(piperazin-1-yl)pyridin-3-yl)-N-(quinoxalin-6-ylmethyl)methanamine (linked compound 38); RNA (79-MER)	79	79	0.3750	0.9494	0.9494	1.81e-28	2hol:A, 7tzt:A

(B)

#	Hit	Length	Strand	E-value	Homologs to hit
1	2hoo:A Crystal structure of an E. coli thi-box riboswitch bound to benfotiamine; thi-box riboswitch	83	+	1.8e-26	
2	2gdi:X Crystal structure of thiamine pyrophosphate-specific riboswitch in complex with thiamine pyrophosphate; TPP riboswitch	80	+	3.8e-26	2gdi:Y, 7tzs:X, 7tzs:Y, 4nya:A, 4nya:B, 7tzr:X, 7tzr:Y
3	7tda:A Crystal structure of the E. coli thiM riboswitch in complex with thiamine pyrophosphate, manganese ions; thiM TPP riboswitch RNA (80-MER)	80	+	3.7e-23	7tdc:A, 7tdb:A, 4nyd:A, 4nyb:A, 4nyc:A
4	2hoj:A Crystal structure of an E. coli thi-box riboswitch bound to thiamine pyrophosphate, manganese ions; thi-box riboswitch	79	+	1.5e-22	4nyg:A
5	7tzu:A Crystal structure of the E. coli thiM riboswitch bound to 1-(4-(piperazin-1-yl)pyridin-3-yl)-N-(quinoxalin-6-ylmethyl)methanamine (linked compound 38); RNA (79-MER)	79	+	7.1e-22	2hol:A, 7tzt:A

Fig 4. FURNA database sequence search results for the *E. coli thiM* riboswitch. (A) Top 5 BLAST search hits. (B) Top 5 Infernal search hits.

(A)

#	Hit	Hit length	Strand	E-value	Homologs to hit
1	2hoo:A Crystal structure of an E. coli thi-box riboswitch bound to benfotiamine; thi-box riboswitch	83	+	1.8e-26	
2	2gdi:X Crystal structure of thiamine pyrophosphate-specific riboswitch in complex with thiamine pyrophosphate; TPP riboswitch	80	+	3.8e-26	2gdi:Y, 7tzs:X, 7tzs:Y, 4nya:A, 4nya:B, 7tzr:X, 7tzr:Y
3	7tda:A Crystal structure of the E. coli thiM riboswitch in complex with thiamine pyrophosphate, manganese ions; thiM TPP riboswitch RNA (80-MER)	80	+	3.7e-23	7tdc:A, 7tdb:A, 4nyd:A, 4nyb:A, 4nyc:A
4	2hoj:A Crystal structure of an E. coli thi-box riboswitch bound to thiamine pyrophosphate, manganese ions; thi-box riboswitch	79	+	1.5e-22	4nyg:A
5	7tzu:A Crystal structure of the E. coli thiM riboswitch bound to 1-(4-(piperazin-1-yl)pyridin-3-yl)-N-(quinoxalin-6-ylmethyl)methanamine (linked compound 38); RNA (79-MER)	79	+	7.1e-22	2hol:A, 7tzt:A
6	2hom:A Crystal structure of an E. coli thi-box riboswitch bound to thiamine monophosphate; thi-box riboswitch	80	+	9.9e-22	
7	7td7:A Crystal structure of an E. coli thiM riboswitch bound to thiamine, manganese ions; thiM riboswitch RNA (78-MER)	78	+	5.7e-21	
8	3k0j:E Crystal structure of the E. coli ThiM riboswitch in complex with thiamine pyrophosphate and the U1A crystallization module; RNA (87-MER)	87	+	4.1e-20	3k0j:F
9	2hok:A Crystal structure of an E. coli thi-box riboswitch bound to thiamine pyrophosphate, calcium ions; thi-box riboswitch	78	+	6.9e-20	
10	8f4o:A Apo structure of the TPP riboswitch aptamer domain; TPP riboswitch aptamer domain	72	+	3e-17	

(B)

#	Hit	Hit length	Strand	E-value	Homologs to hit
1	7tda:A Crystal structure of the E. coli thiM riboswitch in complex with thiamine pyrophosphate, manganese ions; thiM TPP riboswitch RNA (80-MER)	80	+	8.2e-09	7tdc:A, 7tdb:A, 4nyd:A, 4nyb:A, 4nyc:A
2	2hoo:A Crystal structure of an E. coli thi-box riboswitch bound to benfotiamine; thi-box riboswitch	83	+	3.5e-08	
3	2hoj:A Crystal structure of an E. coli thi-box riboswitch bound to thiamine pyrophosphate, manganese ions; thi-box riboswitch	79	+	8.3e-08	4nyg:A
4	2gdi:X Crystal structure of thiamine pyrophosphate-specific riboswitch in complex with thiamine pyrophosphate; TPP riboswitch	80	+	1.8e-07	2gdi:Y, 7tzs:X, 7tzs:Y, 4nya:A, 4nya:B, 7tzr:X, 7tzr:Y
5	2hom:A Crystal structure of an E. coli thi-box riboswitch bound to thiamine monophosphate; thi-box riboswitch	80	+	1.9e-07	
6	7tzu:A Crystal structure of the E. coli thiM riboswitch bound to 1-(4-(piperazin-1-yl)pyridin-3-yl)-N-(quinoxalin-6-ylmethyl)methanamine (linked compound 38); RNA (79-MER)	79	+	2e-07	2hol:A, 7tzt:A
7	2hok:A Crystal structure of an E. coli thi-box riboswitch bound to thiamine pyrophosphate, calcium ions; thi-box riboswitch	78	+	3.7e-07	
8	7td7:A Crystal structure of an E. coli thiM riboswitch bound to thiamine, manganese ions; thiM riboswitch RNA (78-MER)	78	+	5.1e-07	
9	3k0j:E Crystal structure of the E. coli ThiM riboswitch in complex with thiamine pyrophosphate and the U1A crystallization module; RNA (87-MER)	87	+	8.6e-07	3k0j:F
10	8f4o:B Apo structure of the TPP riboswitch aptamer domain; TPP riboswitch aptamer domain	65	+	5.8e-05	

Fig 5. FURNA database sequence search results for TPP riboswitches from *B. subtilis*. (A) Top 5 Infernal search hits for the *ykoF* riboswitch. (B) Top 5 Infernal search hits for the *tenA* riboswitch.

7. Reviewer #3 commented:

FURNA site, it would be nice if dataset.txt had exactly one line per PDB entry, instead of the name running onto the next line. It seems that this is the same breakpoint as in the hover text. It might also be nice if dataset.txt had a more self-documenting name, like FURNA_dataset.txt.

We thank the Reviewer for reporting the issue with “database.txt”. The new FURNA website has renamed “database.txt” into “FURNA_dataset.txt”, where each entry occupies exactly one line.

8. Reviewer #3 commented:

FURNA site, it's a bit strange that the link to the FURNA page about a chain is in the line number. That won't be obvious to people at first, that they should even bother clicking it.

We thank the Reviewer for pointing out this issue, for which we have made two modifications on the FURNA website to improve usability, as shown in following screenshot. First, at the top of the BROWSE page, we explained that users can “Click the entry in the ‘#’ column to view structure details of that RNA chain.” Second, when the mouse is hovered over the first column, there is a hover message saying “Click to view structure details for PDB chain”.

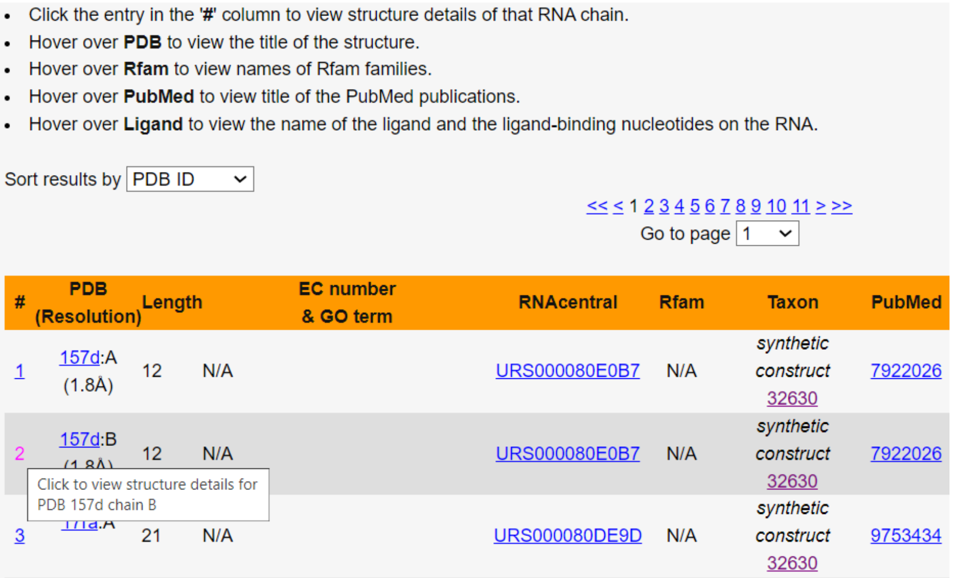
- Click the entry in the '#' column to view structure details of that RNA chain.
- Hover over **PDB** to view the title of the structure.
- Hover over **Rfam** to view names of Rfam families.
- Hover over **PubMed** to view title of the PubMed publications.
- Hover over **Ligand** to view the name of the ligand and the ligand-binding nucleotides on the RNA.

Sort results by PDB ID

<< < 1 2 3 4 5 6 7 8 9 10 11 > >>

Go to page 1

#	PDB (Resolution)	Length	EC number & GO term	RNAcentral	Rfam	Taxon	PubMed
1	157d:A (1.8Å)	12	N/A	URS000080E0B7	N/A	synthetic construct 32630	7922026
2	157d:B (1.8Å)	12	N/A	URS000080E0B7	N/A	synthetic construct 32630	7922026
3	[illegible]:A	21	N/A	URS000080DE9D	N/A	synthetic construct 32630	9753434

Click to view structure details for PDB 157d chain B

9. Reviewer #3 commented:

FURNA site, the mapping of PDB chains to Rfam families is not well enough calibrated, because individual chains often map to multiple Rfam families. For example, bacterial SSU chains map to bacterial, eukaryotic, and archaeal SSU Rfam families, plus many other Rfam families of different molecules. One example is 6h58 chain aa, but there are many others. That's not as helpful as getting the domain right and the molecule right. In dataset.txt, the Rfam hits are listed in lexicographic order, not apparently in order from best hit to worst, so the user can't guess what is the best hit. In some cases, the Rfam site for the family does not map to any chains in PDB, but FURNA maps those

chains to the Rfam family. A more stringent cutoff could be used. Or, once some good hits are found, all worse hits could be dis-regarded.

Line 199, please be clear about the criteria for a hit on an Rfam family, because as mentioned above, hits are reported that don't make much sense.

We thank the Reviewer for identifying the issue, which we agree reduced the utility of Rfam hits in the original version of FURNA. In the version of FURNA used for initial review, the Rfam-PDB matches were identified by cmsearch, which simply shows all Rfam with E-values≤10 in lexicographic order. This causes promiscuity of Rfam matches as identified by the Reviewer. To address this issue, the new version of FURNA features a more stringent and informative Rfam-PDB match procedure, as explained by a new section in the revised Materials and Methods section at page 9:

Rfam family matching
Rfam families are matched to an RNA chain by two approaches. First, we use the Rfam-PDB mapping provided by the Rfam database (https://ftp.ebi.ac.uk/pub/databases/Rfam/CURRENT/Rfam.pdb.gz). Second, if a PDB chain is not included in the mapping file, we search its RNA sequence against the most current version of the Rfam database (Rfam 14.10, with covariance models for 4170 families, https://ftp.ebi.ac.uk/pub/databases/Rfam/CURRENT/Rfam.cm.gz) using Infernal [41]. This Infernal search utilizes the parameters: `cmsearch --cpu 4 -Z 549862.597050 --toponly`, where the search space size parameter 549862.597050 is the same as that used by the Rfam database. Regardless of which approaches are used, Rfam families for an RNA chain are shown in ascending order of their E-values.

Under this new workflow, almost all (98.6%) RNA chains with Rfam in FURNA have only one Rfam family match due to more stringent cmsearch searching parameter.

10. <u>Reviewer #3 commented:</u>

Line 151, with various types of searches going on, and many possible starting points for the searches, it would be good to clarify here, and not just in Table S2, that this is a sequence-based search, and that a genomic sequence of length 200 was input (as opposed to the sequence of some RNA chain from PDB). It would also be good to make clear that FURNA does not allow a user to search an entire genome, but rather the user must locate a short sequence of interest from a genome and then search it against Infernal models derived from PDB chains. As far as I can tell, FURNA is not a tool for annotating full genomes with RNAs whose 3D structures are in PDB.

We thank the Reviewer for the important suggestion. On page 6 of the revised manuscript, we have added the following explanation in the aforementioned paragraph to explain that this is a sequence search, that a genomic sequence of length 200 was used, and that FURNA does not allow search of an entire genome – the user should instead specify a region of interest to query, as we have shown for this case study.

To illustrate FURNA's utility in RNA function annotation, we conducted a case study involving the TPP (thiamin pyrophosphate) binding riboswitches, also known as the THI element or Thi-box riboswitch. This well-known family of riboswitches binds to thiamine pyrophosphate (TPP) to regulate the expression of its downstream gene [36, 37]. In *Escherichia coli*, one such riboswitch is located upstream of the Hydroxyethylthiazole kinase (*thiM*) coding sequence [38, 39] (**Fig. 3**, **Table S2**). We used the 200 nucleotides

immediately upstream of the *thiM* open reading frame as the query sequence to search FURNA.

11. Reviewer #3 commented:

FURNA site, in the 3D structure search results, it would be nice if hovering over a match would show the title of the structure.

We thank the Reviewer for the important suggestion, which is now implemented as shown in the following screen shot. We also explain this new functionality at the header section of the web page.

- Hover over **Hit** or **Homologs to hit** to view the title of the structure.

#	Hit	TM-score normalized by query	TM-score normalized by hit	RMSD	Identity normalized by query	Identity normalized by hit	Identity normalized by aligned length	Length	Aligned length	Homologs to hit
1	5y58:X	1.0000	1.0000	0.00	1.000	1.000	1.000	30	30	
2	1l9a:B	0.3473	0.1162	1.71	0.100	0.024	0.188	126	16	
3	6wlj:A	[illegible]	[illegible]	[illegible]	[illegible]	[illegible]	[illegible]	[illegible]	[illegible]	
4	6mwn:A	[illegible]	[illegible]	[illegible]	[illegible]	[illegible]	[illegible]	[illegible]	[illegible]	
5	1xjr:A	0.3428	0.2795	1.95	0.133	0.085	0.222	47	18	
6	3g8t:P	0.3406	0.1460	2.53	0.400	0.085	0.480	141	25	3g8t:Q, 3g8t:R, 3g8t:S, 2nz4:P, 2nz4:Q, 2nz4:R, 2nz4:S, 3l3c:P, 3l3c:Q, 3l3c:R, 3l3c:S, 3g9c:Q, 3g9c:R, 3g96:P, 3g96:Q, 3g96:R, 3g96:S, 3g8s:P, 3g8s:Q,

CRYSTAL STRUCTURE OF SRP19 IN COMPLEX WITH THE S DOMAIN OF SIGNAL RECOGNITION PARTICLE RNA; signal recognition particle RNA S domain

12. Reviewer #3 commented:

Line 268, please be clear how pseudoknots are removed, because there are different strategies, described in an article by Rob Knight and Sandra Smit, that can lead to different Watson-Crick pairs being removed.

We thank the Reviewer for drawing our attention to this technical detail. The revised Materials and Methods section at page 11 now explains that our pseudoknot removal follows the IL scheme described by Rob Knight and Sandra Smit:

Pseudoknots present in the secondary structures are removed by an incremental length (IL) approach, where nonconflicting paired regions are added one by one, starting with the longest paired region [47].
